# BaiJ and BaiB are key enzymes in the chenodeoxycholic acid 7α-dehydroxylation pathway in the gut microbe *Clostridium scindens* ATCC 35704

**DOI:** 10.1080/19490976.2024.2323233

**Published:** 2024-03-11

**Authors:** Karin Lederballe Meibom, Solenne Marion, Colin Volet, Théo Nass, Eduard Vico-Oton, Laure Menin, Rizlan Bernier-Latmani

**Affiliations:** a Environmental Microbiology Laboratory, École Polytechnique Fédérale de Lausanne (EPFL), Lausanne, Switzerland; b Institute of Chemical Sciences and Engineering, École Polytechnique Fédérale de Lausanne (EPFL), Lausanne, Switzerland

**Keywords:** Clostridium scindens, bile acids, 7α-dehydroxylation, gut microbes, *bai genes*, chenodeoxycholic acid, lithocholic acid, allo-lithocholic acid

## Abstract

Bile acid transformation is a common gut microbiome activity that produces secondary bile acids, some of which are important for human health. One such process, 7α-dehydroxylation, converts the primary bile acids, cholic acid and chenodeoxycholic acid, to deoxycholic acid and lithocholic acid, respectively. This transformation requires a number of enzymes, generally encoded in a bile acid-inducible (*bai*) operon and consists of multiple steps. Some 7α-dehydroxylating bacteria also harbor additional genes that encode enzymes with potential roles in this pathway, but little is known about their functions. Here, we purified 11 enzymes originating either from the *bai* operon or encoded at other locations in the genome of *Clostridium scindens* strain ATCC 35704. Enzyme activity was probed *in vitro* under anoxic conditions to characterize the biochemical pathway of chenodeoxycholic acid 7α-dehydroxylation. We found that more than one combination of enzymes can support the process and that a set of five enzymes, including BaiJ that is encoded outside the *bai* operon, is sufficient to achieve the transformation. We found that BaiJ, an oxidoreductase, exhibits an activity that is not harbored by the homologous enzyme from another *C. scindens* strain. Furthermore, ligation of bile acids to coenzyme A (CoA) was shown to impact the product of the transformation. These results point to differences in the 7α-dehydroxylation pathway among microorganisms and the crucial role of CoA ligation in the process.

## Introduction

Bile acids are synthesized from cholesterol in the liver and stored in the gallbladder until their release into the intestinal tract, where they play an important role in digestion. The primary bile acids produced by humans are cholic acid (CA; 3α,7α,12α-trihydroxy-5β-cholan-24-oic acid) and chenodeoxycholic acid (CDCA; 3α,7α-dihydroxy-5β-cholan-24-oic acid) (the structures of all bile acids discussed in this work are listed in Table S1). Most bile acids are reabsorbed along the small intestine, particularly in the ileum, and are transported back to the liver through the portal vein. This process, known as the enterohepatic circulation, is highly efficient, and only about 5% of the bile acids escape reabsorption.^1,2^

Approximately 400–800 mg of bile acids enter the human large intestine daily and become substrates for microbial transformations resulting in the formation of a range of secondary bile acids.^2^ Bacterial bile salt hydrolases release the primary bile acids from their conjugated taurine- or glycine-groups, and only low amounts of conjugated bile acids are found in fecal samples whereas they dominate throughout the enterohepatic cycle.^1,3^ Other transformations include epimerization, oxidation, and dehydroxylation at C7. The products from 7α-dehydroxylation of CA and CDCA, deoxycholic acid (DCA; 3α,12α-dihydroxy-5β-cholan-24-oic acid) and lithocholic acid (LCA; 3α-hydroxy-5β-cholan-24-oic acid) respectively, are the most abundant secondary bile acids found in human fecal samples.^1,2^

In addition to their function in solubilizing dietary fats, bile acids affect human health by acting as signaling molecules and interacting with various receptors.^4,5^ The 7α-dehydroxylated bile acids DCA and LCA serve as potent agonists for both the nuclear receptor Farnesoid X Receptor (FXR) and the membrane receptor G protein-coupled bile acid receptor (GPBAR1), also called Takeda G-Protein Receptor 5 (TGR5). FXR is the major regulator of bile acid homeostasis but affects other processes, such as glucose and lipid homeostasis, and is also activated by the primary bile acids CA and CDCA. TGR5 is activated mainly by LCA and DCA and is important for various functions, including glucose homeostasis, energy expenditure, and the control of inflammation.^4,5^

**Figure 1.**
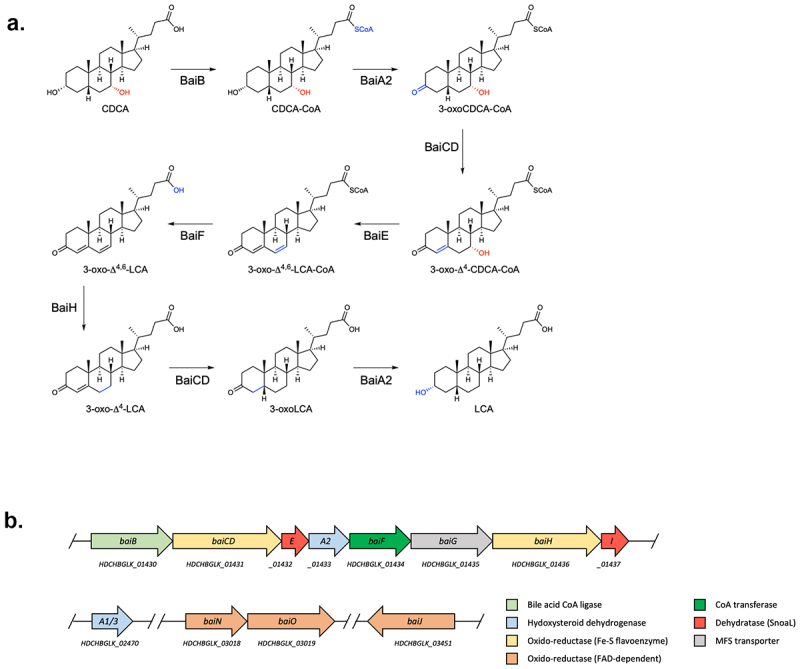
Bile acid 7-dehydroxylation by *C. scindens* ATCC 35704.(a) CA 7α-dehydroxylation proposed by Funabashi and colleagues^9^ (the six-enzyme pathway) projected onto CDCA. (b) *bai* operon along with other genes known or suspected to be involved in bile acid transformations in *C. scindens* ATCC 35704 and the function of the encoded proteins. All enzymes were purified except BaiG.

A bile acid-inducible (*bai*) operon encoding enzymes involved in primary bile acid 7α-dehydroxylation was first identified in *Clostridium scindens* VPI 12708 (formerly *Eubacterium* sp. strain VPI 12708).^6^ A similar operon is found in other strains and other 7α-dehydroxylating species, such as *Extibacter hylemonae* (formerly *Clostridium hylemonae*) and *Peptacetobacter hiranonis* (formerly *Clostridium hiranonis*).^7,8^ The *C. scindens* operon contains eight genes (*baiB-CD-E-A2-F-G-H-I*), encoding one transporter (*baiG*) and 7 enzymes, of which six are sufficient to support 7α-dehydroxylation of CA in enzymatic assays *in vitro* (all but *baiI*).^9^ Bile acid entry into the bacterial cell is facilitated by BaiG,^10^ and is followed by conjugation of coenzyme A (CoA) to CA or CDCA by a CoA ligase encoded by *baiB* ^11^ and two oxidative steps by a 3α-hydroxysteroid dehydrogenase (3α-HSDH; BaiA1 or BaiA2)^9,12^ and a flavin-dependent oxidoreductase (BaiCD),^9,13^ respectively. Following dehydration by BaiE,^14^ formation of DCA is achieved after three reductive steps catalyzed by BaiH (another flavin-dependent oxidoreductase), BaiCD, and BaiA2^9^ (Figure 1a).

In addition to the *bai* genes harbored in the *bai* operon, bile acid 7α-dehydroxylating bacteria contain other genes with potential roles in DCA and LCA production. A *baiJKL* operon is found in some organisms, where *baiK* encodes a bile acid CoA transferase and BaiJ is a flavoprotein that was shown to be involved in the formation of allo-bile acids.^15,16^ Indeed, a homologue of BaiJ (named BaiP by authors) found in other organisms, was likewise shown to be a bile acid 5α-reductase leading to allo-bile acid production.^16^ BaiN is a flavoprotein that may play a role in the reductive part of the 7α-dehydroxylation pathway and the *baiN* gene is consistently found next to *baiO* which encodes a NAD(FAD)-utilizing dehydrogenase for which a role in bile acid metabolism has yet to be shown.^16–18^ Also, many 7-dehydroxylating bacteria harbor 7α- and 12α-HSDHs (hydroxysteroid dehydrogenases) that act on the hydroxyl group found at C7 or C12 (in the case of CA) and furthermore, a gene named *baiP* (different from the *baiJ* homologue mentioned above) was found adjacent to *baiN* and/or *baiO* in some bacteria and hypothesized to encode an exporter of DCA and LCA.^18^

*Clostridium scindens* ATCC 35704^19^ is capable of 7α-dehydroxylation of both human primary bile acids *in vitro*, although the transformation of CDCA to LCA is much less efficient than that of CA to DCA.^20,21^ The reason for the lower amount of LCA is unclear, but could be due to less efficient uptake of the primary bile acid or lower affinity of the enzymes for CDCA than for CA (and/or intermediates), since the expression of *bai* genes is induced in the presence of both CA and CDCA.^21^ The biochemical pathway in its entirety has only been studied with CA as the substrate using purified enzymes *in vitro*, but *bai* operon genes can support 7α-dehydroxylation of CDCA when expressed in *Clostridium sporogenes* ^9^ and the pathway is expected to be equivalent (Figure 1a).

Here, we cloned and purified 11 enzymes from *Clostridium scindens* strain ATCC 35704, both those encoded in the *bai* operon and implicated in the proposed 7α-dehydroxylation pathway^9^ (Figure 1a), and also enzymes suspected to contribute, but encoded by genes that do not pertain to the operon. We used the purified proteins for *in vitro* enzymatic assays under anoxic conditions and found that more than one combination of enzymes leads to the formation of 7α-dehydroxylated bile acids from CDCA. A set of five enzymes is sufficient for CDCA 7α-dehydroxylation, with one enzyme, BaiJ, encompassing an activity that is not harbored in its homologue from *C. scindens* strain VPI 12708.

## Results

### Identification of enzymes supporting 7α-dehydroxylation of CDCA

**Figure 2.**
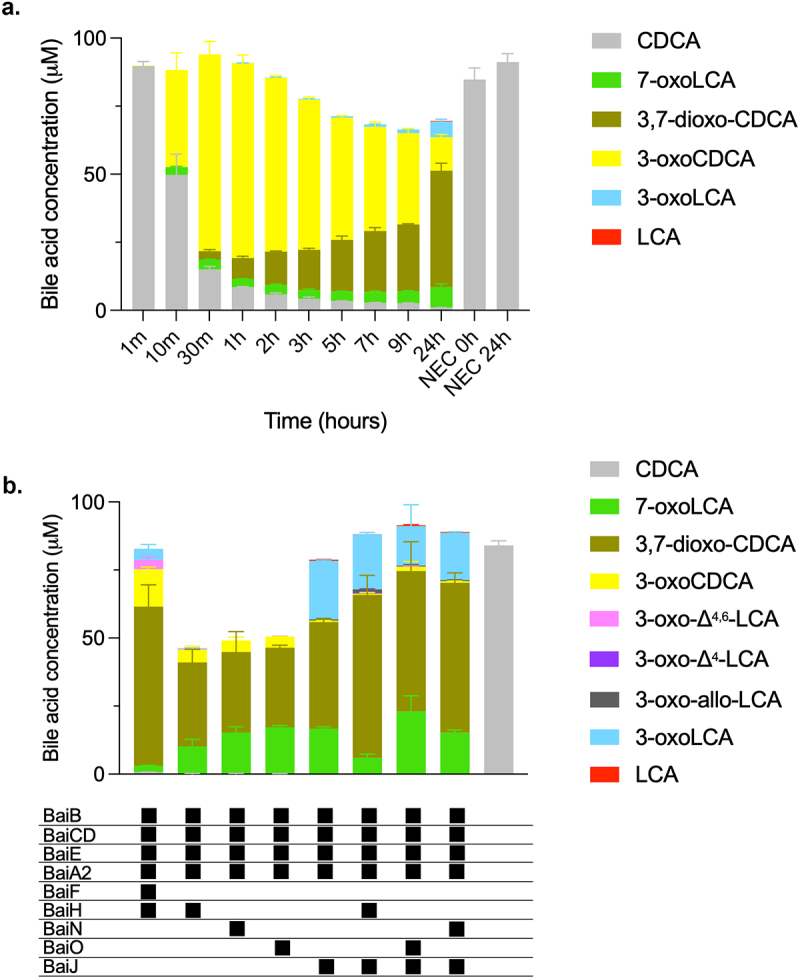
7α-dehydroxylation of CDCA using different enzyme combinations *in vitro*.

To characterize 7α-dehydroxylation of CDCA by *C. scindens* strain ATCC 35704, we performed *in vitro* assays with purified enzymes under anoxic conditions at 37°C and analyzed the bile acid metabolites by liquid chromatography high-resolution mass spectrometry (LC/HRMS). We purified all enzymes encoded in the *bai* operon but were also interested in the role of additional enzymes encoded outside the *bai* operon that potentially contribute to 7α-dehydroxylation (Figure 1b, Figure S1). Therefore, we also purified BaiA1/3, BaiN, BaiO, and BaiJ. We focused on CDCA as the substrate, as the 7α-dehydroxylation pathway of this primary bile acid was not previously characterized in its entirety *in vitro* using purified enzymes. Recently, Funabashi and colleagues^9^ showed that six enzymes encoded in the *bai* operon (BaiB, BaiCD, BaiE, BaiA2, BaiF, and BaiH, hereafter the six-enzyme set), although originating from three different genera (*C. scindens* strain VPI 12708, *P. hiranonis* and *E. hylemonae*), were necessary and sufficient to transform CA into DCA *in vitro*. We probed whether the same set of enzymes from *C. scindens* strain ATCC 35704 converts CDCA to LCA *in vitro* by quantifying the bile acids obtained at different time points (Figure 2a). The six enzymes incubated with 100 μM CDCA only generated a very small amount of LCA (0.24 ± 0.01 μM), and it was only observed after 24 hours incubation. A more significant amount of another 7α-dehydroxylated secondary bile acid, 3-oxoLCA, was produced starting 1 hour after incubation and continued to increase in concentration (reaching 5.68 ± 0.86μM at 24 hours), indicating incomplete conversion to LCA. However, most of the bile acids detected were either in the form of 3-oxoCDCA (initially) or 3,7-dioxo-CDCA (at later time points). The increasing amount of 3,7-dioxo-CDCA shows that most of the 3-oxoCDCA was oxidized at position C7 rather than undergoing 7α-dehydroxylation. Addition of the final enzyme encoded in the *bai* operon, BaiI, had no effect on the bile acid profile obtained after 24 hours of incubation (data not shown).

(a) Bile acids detected after incubation of six-enzyme set (BaiB, BaiCD, BaiE, BaiA2, BaiF, BaiH) or negative control (NEC, no-enzyme control) with 100 μM CDCA at different times. Only CDCA was detected for NEC. Minor amounts of LCA (0.24 ± 0.01 μM) was detected at 24 hours. 3-oxoLCA was detected starting at 1 hour with a maximum at 24 hours (5.68 ± 0.86 μM). No 3-oxoΔ^4^-CDCA, 3-oxo-Δ^4.6^-LCA, or 3-oxo-Δ^4^-LCA was detected. (b) Bile acids detected after 22-24 hours incubation of 100 μM CDCA with the base enzyme set amended with additional oxidoreductase(s), as indicated below the bars. No 3-oxo-Δ^4^-CDCA or allo-LCA were detected in any sample. A minor amount of 3-oxo-Δ^4^-LCA (0.26 ± 0.44 μM) was detected only with one combination of enzymes (BaiB, CD, E, A2, H, J). In addition to 3-oxoLCA (14.51 ± 7.82 μM to 21.44 ± 0.39 μM), minor amounts of 3-oxo-allo-LCA (0.37 ± 0.32 μM to 1.38 ± 0.15 μM) and LCA (0.08 ± 0.02 μM to 0.47 ± 0.03 μM) were detected in all enzyme mixes containing BaiJ. Histograms depict the mean and standard deviation of three assays. Incomplete mass balance is attributed to the formation of CoA conjugates that are not quantifiable due to the absence of standards. The structure of all bile acids discussed here is listed in Table S1. Allo-bile acids were not quantified for data in panel A.

We proceeded to probe whether we could identify a combination of enzymes that converts CDCA to LCA more efficiently. Starting with a base set of four enzymes (hereafter, the base enzyme set) composed of the CoA ligase BaiB, the dehydratase BaiE, the 3α-HSDH BaiA2, and the oxidoreductase BaiCD, we then added other oxidoreductases (BaiH, BaiJ, BaiN, or BaiO). These combinations were tested for their capacity to 7α-dehydroxylate CDCA and were compared to the six-enzyme mix from the *bai* operon (Figure 2b). 7α-dehydroxylated bile acids LCA, 3-oxo-allo-LCA, and 3-oxoLCA (a total of 22.45 μM) were observed when the oxidoreductase BaiJ was added to the base enzyme set, with 3-oxoLCA produced in much larger amounts than the other 7α-dehydroxylated bile acids (21.44 ± 0.39 μM). A very small amount of 3-oxoLCA (0.29 ± 0.04 μM), but no LCA, was detected with BaiH added, and no 7α-dehydroxylation was observed when BaiO or BaiN were the supplemented oxidoreductase. We also assessed whether BaiH, BaiO, or BaiN, when included in addition to BaiJ, would improve the production of 7α-dehydroxylated bile acids, as opposed to when BaiJ alone was present. We did not observe increased amounts of 7α-dehydroxylated bile acids (Figure 2b).

### Five enzymes are sufficient to 7α-dehydroxylate CDCA *in vitro*

**Figure 3.**
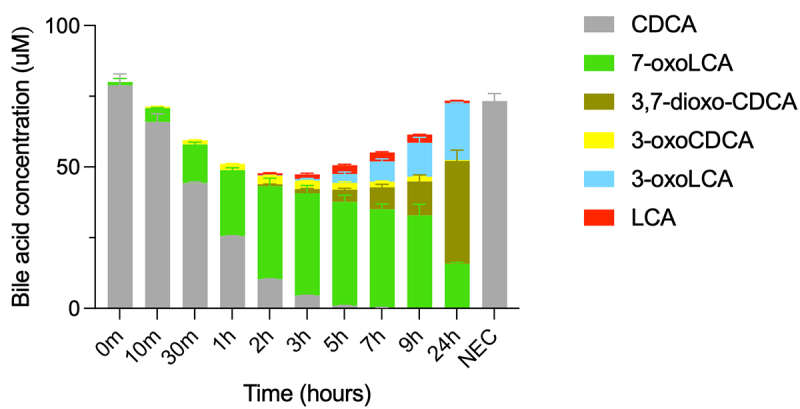
Conversion of CDCA over time with five-enzyme set (BaiB, BaiCD, BaiE, BaiA2, BaiJ).

Next, we examined CDCA conversion with the set of enzymes identified to be the most efficient at producing 7α-dehydroxylated bile acids, namely the mix of BaiB, BaiCD, BaiE, BaiA2, and BaiJ (hereafter, the five-enzyme set), resulting in 7α-dehydroxylation over a period of 24 hours (Figure 3). The two 7α-dehydroxylated secondary bile acids, 3-oxoLCA and LCA, were detected starting at 2 hours, with the concentration of 3-oxoLCA increasing over the entire experiment (20.05 ± 0.32 μM at 24 hours) and the concentration of LCA increasing until 5–9 hours and then decreasing between 9 and 24 hours. The continuous increase in 3-oxoLCA, alongside the decrease in LCA at 24 hours suggests that re-oxidation of LCA to 3-oxoLCA occurred. No allo-bile acids (3-oxo-allo-LCA or allo-LCA) were observed in this experiment, whereas a minor amount was observed in the previous experiment (Figure 2b). As seen with the six-enzyme set (Figure 2a), we observed a large proportion of bile acids that were oxidized at position C7, either in the form of 7-oxoLCA or 3,7-dioxo-CDCA. We did not observe all of the proposed intermediates of the pathway, as no 3-oxo-Δ^4^-CDCA, 3-oxo-Δ^4,6^-LCA or 3-oxo-Δ^4^-LCA was detected at any time (Figure 3). This may be due to either to the fact that they are short-lived or that they are found only as CoA conjugates. The latter interpretation is parsimonious, as the bile acid mass balance was not achieved in the middle of the experiment, from 10 min to 9 hours (Figure 3), while it was at the late time point (24 hours). Indeed, while bile acid CoA conjugates could be detected throughout the experiment based on either enzymatically synthesized standards or their mass (Figure S2), their concentrations could not be quantified due to the absence of commercial standards. Most of the intermediates were identified, including CDCA-CoA, 3-oxoCDCA-CoA, 3-oxo-Δ^4,6^-LCA-CoA, 3-oxo-Δ^4^-LCA-CoA, and LCA-CoA (Figure S2).

Bile acids detected after incubation of five-enzyme set with 100 μM CDCA and sampled at various times. Only CDCA was detected for no-enzyme control (NEC, 24 hours). LCA and 3-oxoLCA were detected starting at 2 hours. No 3-oxo-Δ^4^-CDCA, 3-oxo-Δ^4,6^-LCA, 3-oxo-Δ^4^-LCA or allo-bile acids were detected. Histograms depict the mean and standard deviation of three assays. Incomplete mass balances (between 10 minutes and 9 hours) are attributed to the formation of CoA conjugates that are not quantifiable due to the absence of standards. CoA conjugated bile acids are shown in Figure S2. The structure of all bile acids discussed here is listed in Table S1.

A paralogue of BaiA2, encoded by the *baiA1/3* gene, is present in the genome of *C. scindens* strain ATCC 35704. As the sequences of the two proteins are about 90% identical, we hypothesized that BaiA1/3 could substitute for BaiA2 in the five-enzyme mix. As anticipated, replacing BaiA2 with BaiA1/3 resulted in 7α-dehydroxylation and the bile acid profile obtained was very similar to that obtained when BaiA2 was included (Figure S3), showing that BaiA2 and BaiA1/3 are interchangeable.

### Heterologous expression of *bai* genes in *E. coli* supports 7α-dehydroxylation of CDCA

**Figure 4.**
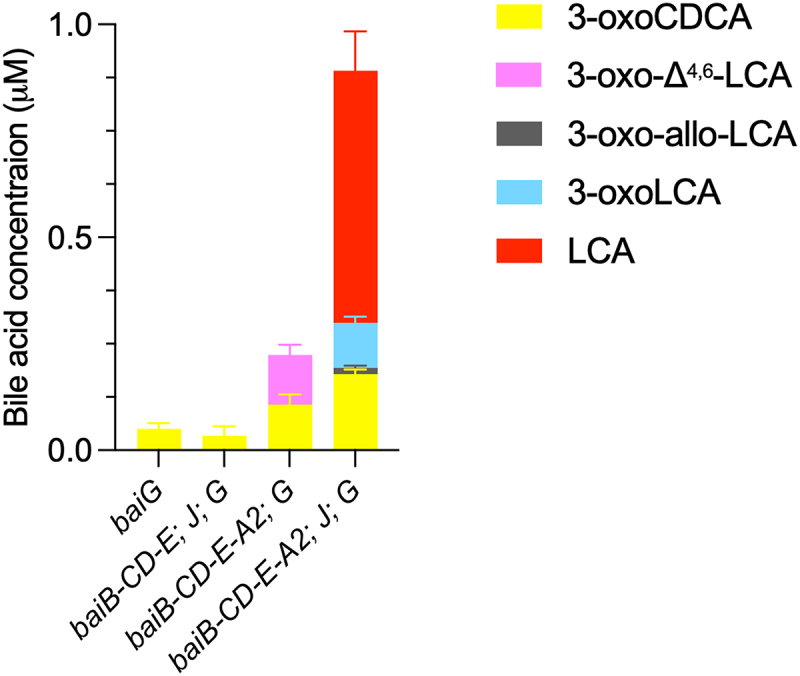
Conversion of CDCA by *E. coli* expressing *bai* genes.

No genetic system has been established in *C. scindens* to date, preventing us from directly assessing the involvement of individual genes in the pathway. Instead, we opted to engineer several *E*. *coli* strains expressing various *bai* genes (Figure S4A) to support the purified enzyme findings. In addition to the five-enzyme set, shown above to catalyze 7α-dehydroxylation *in vitro*, we also introduced the gene encoding the bile acid transporter BaiG. When only this gene is present, *E. coli* transformed a large fraction of CDCA to 7-oxoLCA (Figure S4B), indicating the presence of a 7α-HSDH in this bacterium, as has been reported before.^22^ However, when all five genes (*baiB-baiCD-baiE-baiA2; baiJ*) are present in addition to *baiG*, the *E. coli* strain converted a small fraction of CDCA to 3-oxoLCA and LCA and a very small amount of 3-oxo-allo-LCA (Figure 4). In the absence of either *baiA2* or *baiJ*, no 7α-dehydroxylation took place. These data support the finding with the purified enzymes that BaiB, BaiCD, BaiE, BaiA2, and BaiJ (the five-enzyme set) are sufficient to obtain conversion of CDCA to 7α-dehydroxylated secondary bile acids.

Concentration of selected bile acids detected after incubation of engineered *E. coli* strains with 200 μM CDCA and sampled after 24 hours. Only detected intermediates of the 7α-dehydroxylation pathway are shown to allow visualization of bile acids found in low amounts. Concentrations of all detected bile acids are shown in Figure S4B. The *bai* genes in each strain are shown below bars. The last bar corresponds to the five-enzyme set plus BaiG. Histograms depict the mean and standard deviation of three assays. The structure of all bile acids discussed here is listed in Table S1.

### BaiJ is involved in the reductive part of the pathway

**Figure 5.**
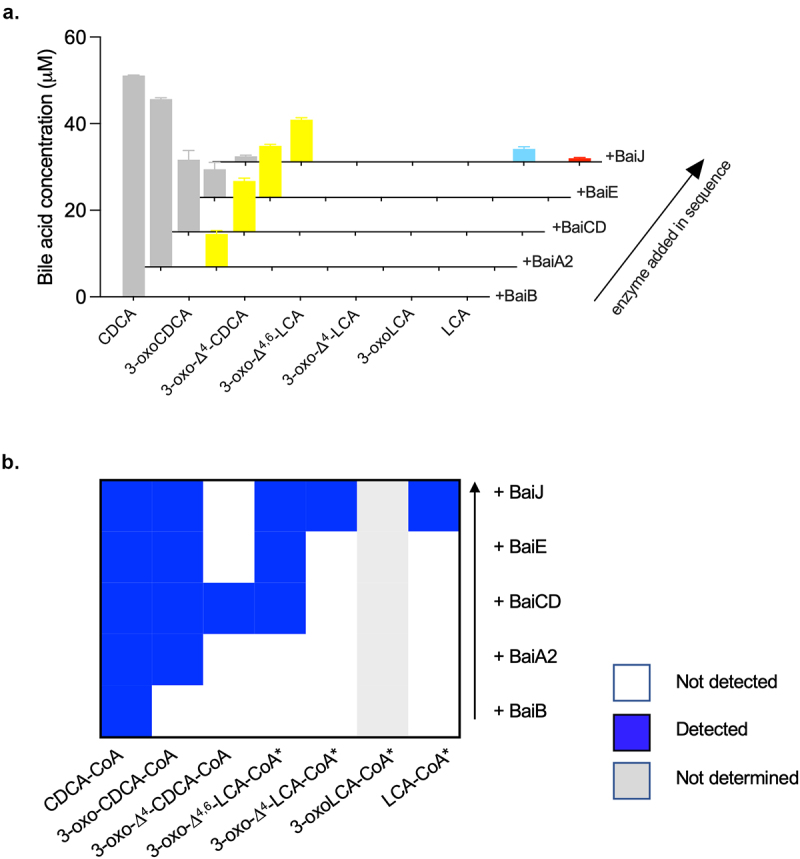
Conversion of CDCA during sequential addition of five-enzyme set enzymes.

To detect intermediates of the 7α-dehydroxylation pathway and evaluate the involvement of individual enzymes in specific steps, we added single enzymes from the five-enzyme set to CDCA, one at a time, in the sequence in which we expected them to act. Initially, CDCA was incubated with BaiB, followed by the addition of BaiA2, of BaiCD, of BaiE and finally, addition of BaiJ. Before supplementation with each enzyme, a sample was taken and the bile acids quantified (Figure 5a). 3-oxoCDCA appeared as expected after BaiA2 was added to the reaction, and the concentration increased during the experiment. However, we did not detect the intermediates 3-oxo-Δ^4^-CDCA, 3-oxo-Δ^4,6^-LCA or 3-oxo-Δ^4^-LCA, suggesting that these only exist as CoA conjugates. In agreement with this inference, we observed 3-oxo-Δ^4^-CDCA-CoA and a bile acid-CoA compound with a mass corresponding to 3-oxo-Δ^4,6^-LCA-CoA after addition of BaiCD (Figure 5b). No new bile acid-CoA compounds were observed after the addition of BaiE, but two novel bile acid-CoAs with masses corresponding to 3-oxo-Δ^4^-LCA-CoA and LCA-CoA were detected after addition of BaiJ. In addition, we observed CDCA-CoA at all steps and 3-oxoCDCA-CoA at all steps after adding BaiA2. These data corroborate that BaiB ligates CoA to CDCA, followed by oxidation at position C3 by BaiA2 forming 3-oxoCDCA-CoA, but also 3-oxoCDCA from unconjugated CDCA. The next steps are catalyzed by BaiCD and BaiE (oxidation followed by dehydration), but we only detected these intermediates in the form of CoA conjugates. Finally, we only observed the bile acids 3-oxo-LCA and LCA and CoA compounds with masses corresponding to 3-oxo-Δ^4^-LCA-CoA and LCA-CoA after the addition of BaiJ (we could not determine whether 3-oxoLCA-CoA was present). This strongly suggests that BaiJ participates in the reductive steps of the pathway.

(a) Bile acid concentrations after incubation of 100 μM CDCA with indicated enzyme(s) for 90 min (150 min for BaiB) after which the next enzyme was added. Only bile acids in the proposed pathway are shown, but 7-oxoLCA and 3,7-dioxo-CDCA were also detected. Only CDCA was detected for no-enzyme control (not shown). No 3-oxo-Δ^4^-CDCA, 3-oxo-Δ^4,6^-LCA, 3-oxo-Δ^4^-LCA or allo-bile acids were detected. Histograms depict the mean and standard deviation of three assays. The structure of the bile acids discussed here is listed in Table S1. (b) CoA conjugated bile acids detected at each step. CoA intermediates with * are only identified by their mass, whereas the others were identified on the basis on enzymatically produced standards. 3-oxoLCA-CoA could not be detected due a technical problem.

### BaiJ catalyzes the first reductive step of the pathway

**Figure 6.**
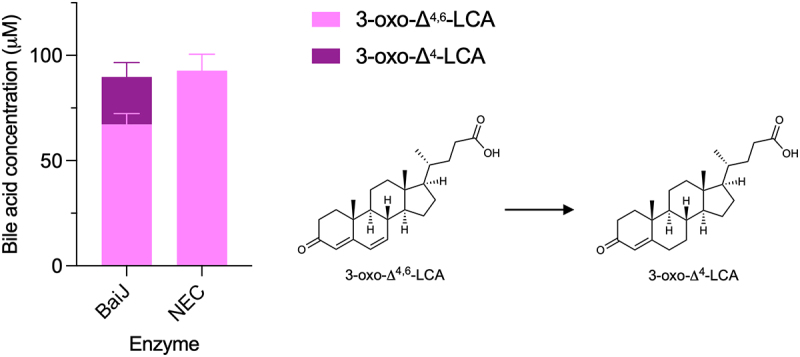
First reductive step in 7α-dehydroxylation pathway.

Based on the CA pathway, the reductive steps are expected to proceed via the reduction of 3-oxo-Δ^4,6^-LCA to 3-oxo-Δ^4^-LCA and then to 3-oxoLCA and LCA (Figure 1a). The five-enzyme set produced both 3-oxoLCA and LCA (Figure 3). Therefore, we hypothesized that BaiJ is involved in the first reductive step and perhaps the second, as it was shown to be necessary to produce LCA. To assess this activity, the enzyme was incubated with 3-oxo-Δ^4,6^-LCA and was found to reduce it to 3-oxo-Δ^4^-LCA (Figure 6). In the model of the pathway based on the six-enzyme set, this first reductive step is catalyzed by BaiH.^9^ Thus, our data reveal that BaiJ can replace BaiH for this step in *C. scindens* strain ATCC 35704 and is responsible for the initiation of the reductive part of the pathway with the five-enzyme set.

Bile acid concentrations after incubation of 3-oxo-Δ^4,6^-LCA with BaiJ from strain ATCC 35704 for 3 hours. A minor amount of 3-oxo-Δ^4^-CDCA was seen in both samples (2.41 ± 0.07 μM and 2.21 ± 0.08 μM), indicating a slight enzyme-independent transformation (not shown in graph). Allo-bile acids were not assessed as they are not relevant for the first reductive step but a minor amount of a bile acid observed with BaiJ (not shown in graph and assigned as 3-oxo-LCA in data file), is believed to be 3-oxo-allo-LCA. Histograms depict the mean and standard deviation of three assays. Structures of bile acids are shown on the right.

### The second reductive step requires CoA ligation

**Figure 7.**
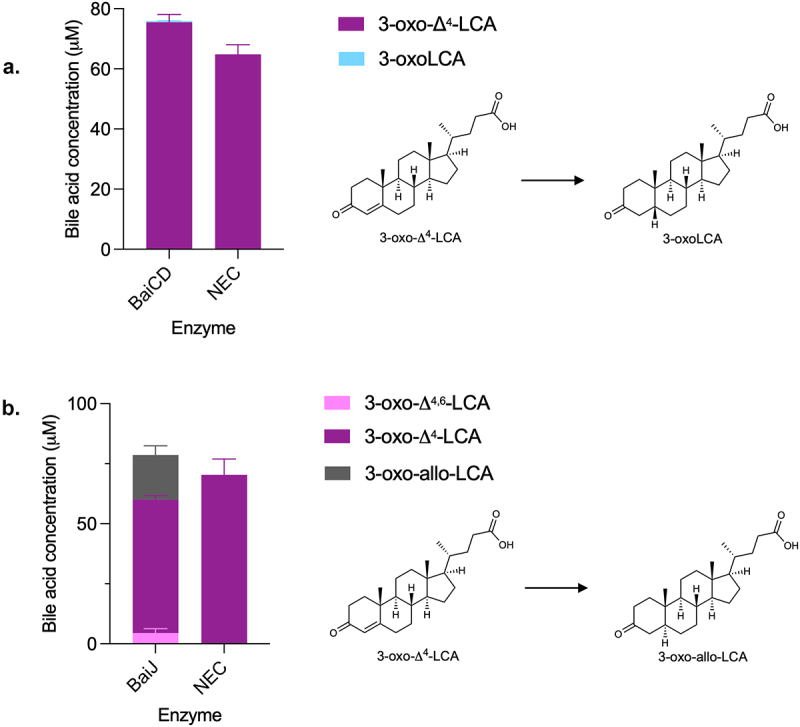
Second reductive step in 7α-dehydroxylation pathway.

BaiCD has previously been shown to catalyze the second reductive step, the conversion of 3-oxo-Δ^4^-DCA to 3-oxoDCA, in the CA pathway.^9^ We hypothesized that it would exhibit the same activity in the CDCA 7α-dehydroxylation pathway. Surprisingly, a very small amount of 3-oxoLCA (0.40 ± 0.04 μM) was produced from 3-oxo-Δ^4^-LCA after 24 hours incubation with BaiCD (Figure 7a).

(a) 3-oxo-Δ^4^-LCA was incubated with BaiCD for 24 hours and bile acids quantified. Only a small amount of 3-oxoLCA (0.40 ± 0.04 μM) was detected with BaiCD. A slight contamination with 3,7-dioxoCDCA was observed in both assays (not shown). (b) 3-oxo-Δ^4^-LCA was incubated with BaiJ for 3 hours and bile acids quantified. Only 3-oxo-Δ^4,6^-LCA, 3-oxo-Δ^4^-LCA and 3-oxo-allo-LCA were detected. Histograms depict the mean and standard deviation of three assays. Structures of bile acids are shown on the right.

Therefore, we probed the activity of BaiJ with 3-oxo-Δ^4^-LCA. A recent study has shown that BaiJ from *C. scindens* strain VPI 12708 and from strain ATCC 35704 (named BaiP by authors) formed allo-bile acids (3-oxo-allo-LCA and allo-LCA) from 3-oxo-Δ^4^-LCA, when expressed in *E. coli* either alone or in combination with BaiA1.^16^ Our data confirmed that BaiJ reduces 3-oxo-Δ^4^-LCA and forms 3-oxo-allo-LCA as the only reduced product (Figure 7b). In addition, it was also capable of oxidizing 3-oxo-Δ^4^-LCA to 3-oxo-Δ^4,6^-LCA, a reversal of the first reductive step for which BaiJ is responsible in the five-enzyme set (Figure 7b).

The formation of allo-bile acids from the reduction of 3-oxo-Δ^4^-LCA by BaiJ was surprising given that very little (or no) allo-bile acids were observed during CDCA 7α-dehydroxylation with the five-enzyme set or in examination of a previous experiment with whole cells.^21^ When we did observe allo-bile acids with the five-enzyme set, only 3-oxo-allo-LCA was detected, and it was found in much lower concentrations than 3-oxoLCA (15- to 46-fold less) (Figure 2b, Figure S3).

**Figure 8.**
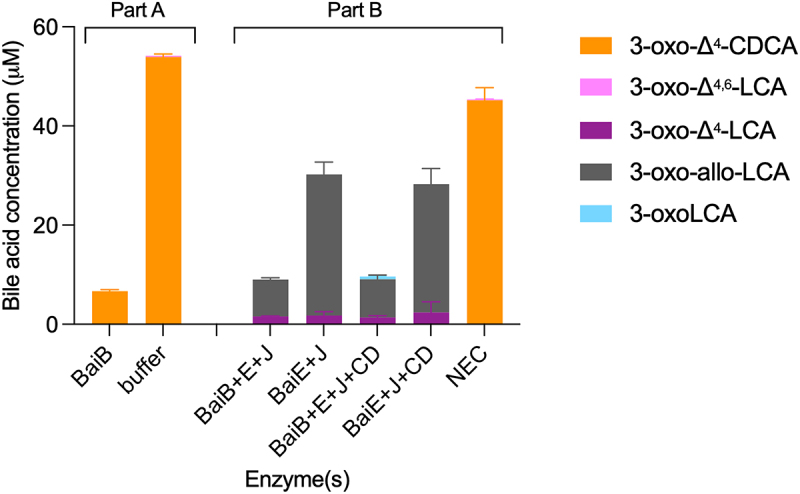
Second reductive step with CoA-ligated bile acids.

A significant difference between CDCA 7α-dehydroxylation with the five-enzyme set and the assays performed with single enzymes and 3-oxo-Δ^4^-LCA, is the presence of BaiB in the former, which allows for the formation of CoA-conjugated bile acids. To explore whether CoA conjugation plays a role in the formation of allo-bile acids, we compared the products of 7α-dehydroxylation in the presence and absence of CoA-conjugated bile acids, i.e., with and without BaiB. It was not possible to use CDCA as the substrate for this experiment, as omitting BaiB essentially blocks the 7α-dehydroxylation pathway (see Figure 1A), and only the early intermediate 3-oxoCDCA as well as the 7-oxidized bile acids (7-oxoLCA, 3,7-dioxo-CDCA) are formed in significant amounts (Figure S5A). Instead, we opted to use the 3-oxo-Δ^4^-CDCA intermediate, as it is a substrate for BaiB-dependent CoA ligation, which is the case neither for 3-oxo-Δ^4,6^-LCA nor 3-oxo-Δ^4^-LCA intermediates (data not shown). 3-oxo-Δ^4^-CDCA was incubated in buffer with CoA alone or containing BaiB for 18 hours (Figure 8, part A) and, as expected, the concentration of 3-oxo-Δ^4^-CDCA decreased significantly in the reaction with BaiB, indicating conjugation to CoA. We then added BaiE+BaiJ or BaiE+BaiJ+BaiCD to either pre-incubation condition and quantified the bile acids after 7 hours of incubation (Figure 8, Part B). In all incubations, we only recovered part of the total bile acids present; a total of about 45 μM bile acids were found in the no-enzyme control (NEC) compared to a total of 9–10 μM with BaiB and 28–30 μM without BaiB. In the former case, we expect CoA-conjugated forms to be present (but not quantifiable). However, we could not identify them because it is not possible to discriminate between 3-oxo-allo-LCA-CoA and 3-oxoLCA-CoA as there are no standards for either. In the assays without BaiB, we also observed a lower concentration than in the NEC. This may be due to the properties of some of the intermediates, as we have observed that both 3-oxo-allo-LCA and 3-oxoLCA likely adsorb onto the surface of the assay tube. As neither BaiA2 nor BaiA1/3 were included, we expected no production of LCA or allo-LCA, which was indeed the case. In all cases, a significant amount of 3-oxo-allo-LCA was produced, but more so in the assays without BaiB pre-incubation. A small amount of 3-oxoLCA (0.53 ± 0.02 μM) was produced in the BaiB pre-incubation case, but only when BaiCD was present in addition to BaiE and BaiJ. Together, these data support a scenario where BaiJ produces 3-oxo-allo-LCA from 3-oxo-Δ^4^-LCA in the absence of CoA and where 3-oxoLCA is only formed when CoA-conjugated bile acids and BaiCD are present. This suggests that BaiCD reduces 3-oxo-Δ^4^-LCA-CoA.

Part A: 3-oxo-Δ^4^-CDCA was incubated for 18 hours in buffer containing CoA and with or without BaiB and bile acids quantified (only 3-oxo-Δ^4^-CDCA was detected, apart from a minor amount of 3-oxo-Δ^4,6^-LCA seen without BaiB and in the NEC, possibly due to enzyme-independent dehydration). Part B: Each incubation was amended with additional enzymes (BaiE+BaiJ or BaiE+BaiJ+BaiCD) and cofactors and bile acids were quantified after 7 hours. 3-oxoLCA (0.53 ± 0.02 μM) was only detected in the assay with BaiB+BaiE+BaiJ+BaiCD. Histograms depict the mean and standard deviation of three assays. The structure of bile acids discussed here is listed in Table S1.

### BaiJ from *C. scindens* strain VPI 12708 differs from BaiJ from strain ATCC 35704

**Figure 9.**
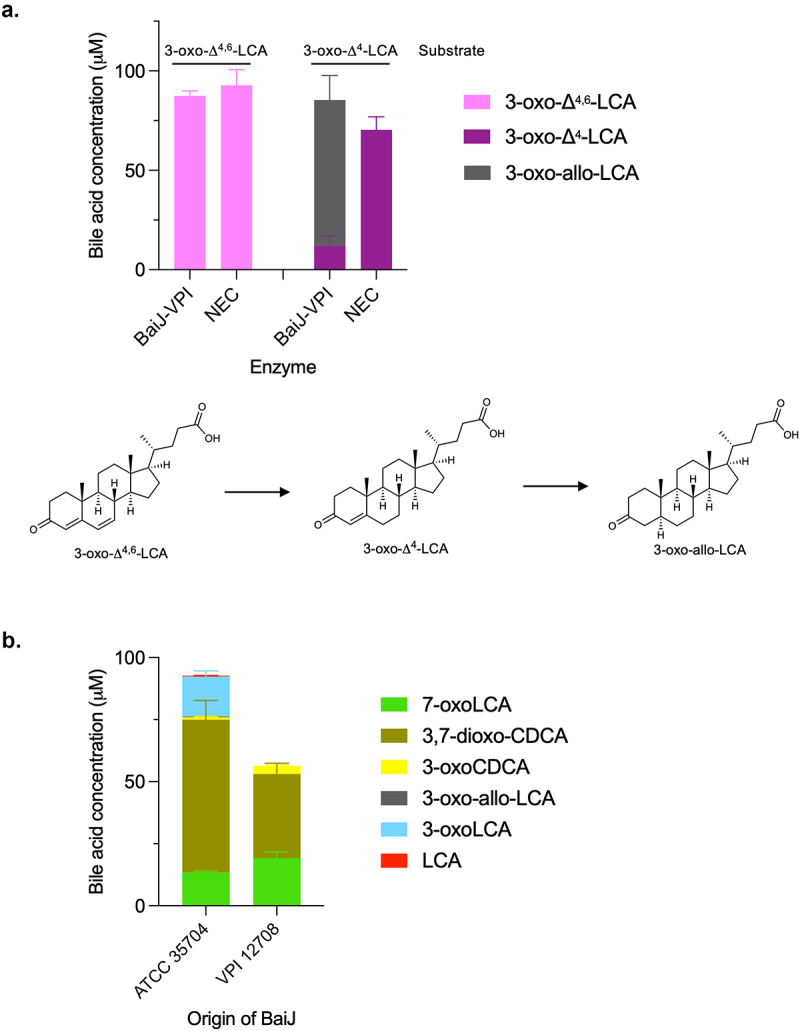
BaiJ from *C. scindens* strain VPI 12,708 cannot initiate reductive part of 7α-dehydroxylation pathway.

BaiJ from *C. scindens* ATCC 35704 is only approximately 47% identical to the *C. scindens* VPI 12708 BaiJ protein (hereafter VPI-BaiJ), which has also been shown to form allo-bile acids from 3-oxo-Δ^4^-LCA.^16^ However, it is unclear whether VPI-BaiJ performs the first reductive step in the 7α-dehydroxylation pathway (3-oxo-Δ^4,6^-LCA to 3-oxo-Δ^4^-LCA) as we reported for BaiJ from the ATCC 35704 strain (Figure 6). We found that VPI-BaiJ was unable to reduce 3-oxo-Δ^4,6^-LCA to 3-oxo-Δ^4^-LCA, but was able to reduce 3-oxo-Δ^4^-LCA to 3-oxo-allo-LCA (Figure 9a) as previously reported.^16^ Since BaiJ in the five-enzyme set is responsible for initiating the reductive part of the pathway, we expected that the VPI-BaiJ would not replace the ATCC 35704 BaiJ in the five-enzyme set. This was indeed the case as no 7α-dehydroxylated bile acids (3-oxoLCA or LCA) were detected when the five-enzyme set incubated with CDCA contained VPI-BaiJ instead of BaiJ from the ATCC 35704 strain (Figure 9B).

(a) Bile acid concentrations after incubation of 100 μM 3-oxo-Δ^4,6^-LCA or 3-oxo-Δ^4^-LCA with BaiJ from *C. scindens* VPI 12708 for 3 hours. Structures of bile acids for the reactions are shown below graph. A minor amount of 3-oxo-Δ^4^-CDCA (2.21 ± 0.08 μM) was observed in the NEC with 3-oxo-Δ^4,6^-LCA as substrate (not shown in graph). (b) Five-enzyme set with BaiJ from either strain ATCC 35704 or VPI 12708 (as indicated) incubated for 24 hours with 100 μM CDCA. No 3-oxo-allo-LCA, 3-oxoLCA or LCA was produced with BaiJ from strain VPI 12708. Only CDCA was detected in the NEC (not shown). Histograms depict the mean and standard deviation of three assays.

### BaiA2 or BaiA1/3 catalyzes the last step in both directions

**Figure 10.**
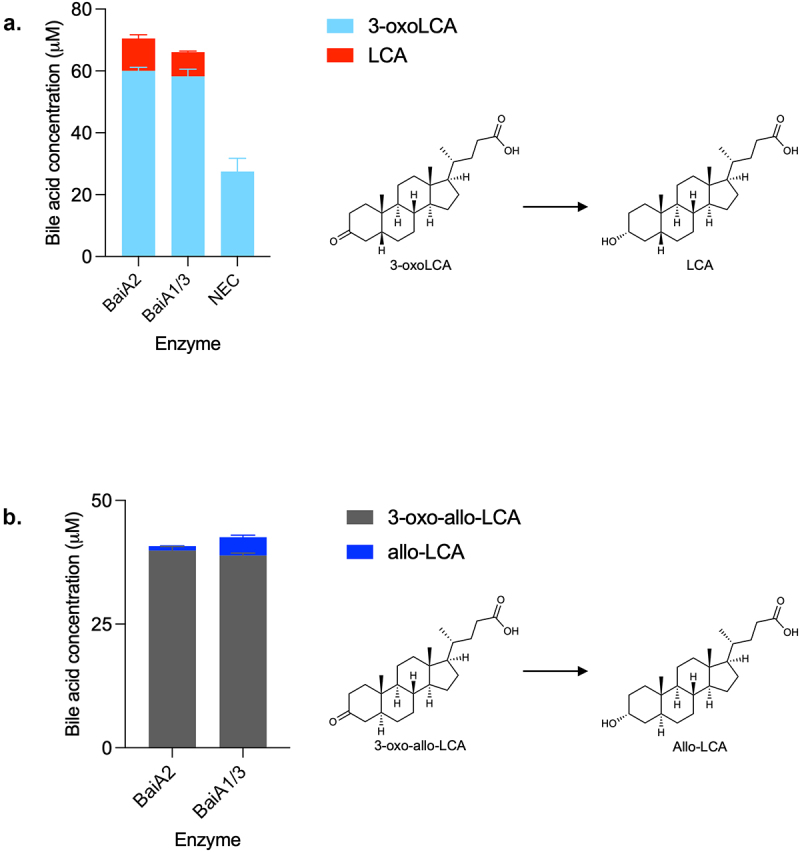
Third reductive step in 7α-dehydroxylation pathway.

The third reductive and last step in the 7α-dehydroxylation of CDCA is the conversion of 3-oxoLCA to LCA, which has previously been attributed to BaiA2 in the case of DCA production.^9^ Accordingly, we found that both BaiA2 and BaiA1/3 reduce 3-oxoLCA to LCA (Figure 10a). As mentioned earlier, in our experiment assessing the 7α-dehydroxylation of CDCA with the five-enzyme set over time, we observed that the amount of LCA initially increased, followed by a decrease, whereas 3-oxoLCA continuously increased in concentration (Figure 3). This indicates re-oxidation of LCA to 3-oxoLCA and indeed, we observed that both BaiA2 and BaiA1/3 support the reaction in the oxidative direction as well (Figure S6).

Bile acid concentrations after incubation of (a) 3-oxoLCA for 3 hours or (b) 3-oxo-allo-LCA for 1 hour with indicated enzymes. Only 3-oxo-allo-LCA was detected in the NEC (panel B, not shown) after 24 hours incubation. Histograms depict the mean and standard deviation of three assays. Structures of bile acids are shown on the right.

Furthermore, we also found that both BaiA2 and BaiA1/3 were capable of reducing 3-oxo-allo-LCA to allo-LCA (Figure 10b) and of oxidizing allo-LCA to 3-oxo-allo-LCA (data not shown).

## Discussion

Most studies on the biochemical pathway of 7α-dehydroxylation have focused on the genes encoded in the canonical *bai* operon, *baiB-CD-E-A2-F-G-H-I* and on *C. scindens* strain VPI 12708. We purified all enzymes encoded in the *bai* operon, as well as those encoded by the genes *baiJ, baiN, baiO* and *baiA1/3* from *C. scindens* strain ATCC 35704 and used anoxic *in vitro* enzyme assays to probe the role of the various enzymes in CDCA 7α-dehydroxylation. While the six-enzyme set previously identified to 7α-dehydroxylate CA supports limited 7α-dehydroxylation, we found that other enzyme combinations, all including BaiJ, enhanced the overall yield. Indeed, a five-enzyme set (BaiB, CD, E, A2 and J), 7α-dehydroxylates CDCA more efficiently (Figure 3) than the six-enzyme set (BaiB, CD, E, A2, F, H) previously invoked for strain VPI 12708 (Figure 2a).^9^ In addition, we found that BaiA2 encoded in the *bai* operon can be replaced by BaiA1/3 encoded at another locus (Figure S3). BaiA1/3 and BaiA2 are approximately 90% identical and it is therefore not surprising that they perform similar roles. *C. scindens* VPI 12708 encodes two BaiA proteins as the ATCC 35704 strain,^16,23,24^ but not all 7α-dehydroxylating species contain two *baiA* genes. *Extibacter hylemonae* DSM 15053 only harbors a single *baiA* gene that is located adjacent to the *baiJKL* cluster and not in the *bai* operon, whereas *Peptacetobacter hiranonis* DSM 13275 and *Proteocatella sphenisci* DSM 23131 only harbor a single *baiA* gene, located within the *bai* operon.^16,25^

The temporal bile acid profiles during CDCA 7α-dehydroxylation differ between the five-enzyme and the six-enzyme set (Figures 3 and 2a). In the latter case, rapid appearance of predominantly 3-oxoCDCA was observed, as opposed to 7-oxoLCA for the five-enzyme set. However, after 24 hours, both reactions contained 3,7-dioxo-CDCA as the major bile acid, indicating that one or more enzymes can oxidize the hydroxyl group at C7. We found that both BaiCD and BaiJ were capable of this activity (Figure S7), and the presence of both in the five-enzyme set likely favored rapid oxidation at C7, resulting in limited 7α-dehydroxylation. Indeed, at 24 hours almost all of the bile acids were found in the form of either 7-dehydroxylated or 7-oxidized species, with only a very minor amount of 3-oxoCDCA left (Figure 3). For the six-enzyme set, a significant amount of 3-oxoCDCA remained at 24 hours, which could be transformed into 3,7-dioxo-CDCA or 7α-dehydroxylated (Figure 2a).

**Figure 11.**
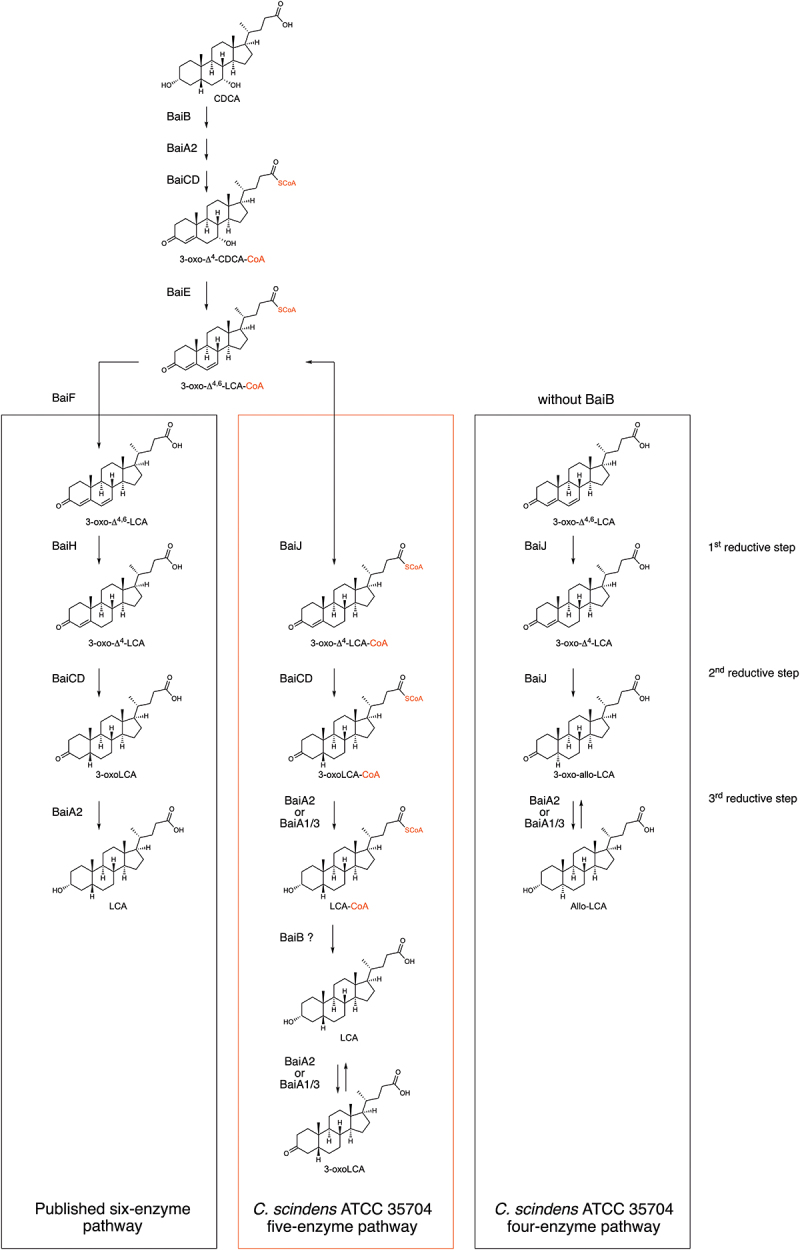
Model of CDCA 7α-dehydroxylation in *C. scindens* ATCC 35704.

The differences between these two enzyme combinations leading to 7α-dehydroxylation in *C. scindens* ATCC 35704, are (a) the substitution of BaiH in the six-enzyme set with BaiJ, and (b) the presence of a CoA transferase (BaiF) in the six-enzyme set. By analogy to the published CA pathway, BaiH is expected to initiate the reductive branch in the CDCA pathway,^9^ converting 3-oxo-Δ^4,6^-LCA to 3-oxo-Δ^4^-LCA (Figures 1a and 11). Here, we show that BaiJ from strain ATCC 35704 performs this activity (Figure 6), whereas BaiH from the ATCC strain has almost no activity (data not shown). This BaiJ activity seems to be restricted to some organisms, as the protein from *C. scindens* strain VPI 12708 is not able to reduce 3-oxo-Δ^4,6^-LCA, and hence, a five-enzyme set including BaiJ from strain VPI 12708 is not functional (Figure 9b). BaiJ homologues are found in many organisms and a recent phylogenetic analysis showed that BaiJ from *C. scindens* VPI 12708 is found in a separate sub-cluster from the *C. scindens* ATCC 35704 BaiJ.^16^ It was found that BaiJ from strain ATCC 35704 clustered with proteins from other *C. scindens* strains as well as from *Pe. hiranonis* and *Pr. sphenisci*, whereas BaiJ from the VPI 12708 strain clustered with proteins from *Dorea* and Oscillospiraceae among others.^16^ Interestingly, *Pe. hiranonis* harbors two *baiJ* genes and one of the two proteins clusters with BaiJ from *C. scindens* VPI 12708, while the other clusters with the ATCC 35704 enzyme. These results suggest that there may be two distinct pathways for CDCA 7α-dehydroxylation, one involving BaiJ in the first reductive step and the other involving BaiH (or another oxidoreductase).

CDCA is transformed to 3-oxo-Δ^4^-CDCA-CoA by BaiB, BaiA2, and BaiCD, followed by dehydration catalyzed by BaiE to form 3-oxo-Δ^4,6^-LCA-CoA, as described previously.^9^ The next steps in the published 7α-dehydroxylation pathway based on *bai* operon-encoded six-enzyme set are shown on the left.^9^ In our model of CDCA 7α-dehydroxylation with the *C. scindens* ATCC 35704 five-enzyme set (middle panel), the first reductive step directly follows the dehydration step and is catalyzed by BaiJ (and is reversible). The second reductive step is most likely catalyzed by BaiCD (as BaiCD is necessary to form 3-oxoLCA when starting from 3-oxo-Δ^4^-CDCA, Figure 8). The final reductive step can be catalyzed by either BaiA2 or BaiA1/3. CoA is expected to be lost only after the third reductive step (as we observed the presence of LCA-CoA), resulting in production of LCA. The final product LCA may be re-oxidized to 3-oxo-LCA by either BaiA2 or BaiA1/3. Without BaiB (right panel), a minor fraction of bile acids may proceed through the pathway and unconjugated 3-oxo-Δ^4,6^-LCA enters the reductive branch where BaiJ catalyzes both the first and second reductive step resulting in the formation of 3-oxo-allo-LCA, with the possibility of further reduction to allo-LCA by either BaiA2 or BaiA1/3.

Expression of genes in the *bai* operon is induced by CA in various organisms,^6,7,21,23,25^ but less is known about genes encoded at other loci. It has been observed that expression of *baiA1/3* and *baiJ* in *C. scindens* strain ATCC 35704 is induced by CA, whereas *baiN* is expressed at the same level with or without CA.^23^ It was recently found that the *baiJ* gene in *C. scindens* strain VPI 12708 is not induced by CA (or other primary bile acids) as opposed to *baiJ* in strain ATCC 35704,^21^ which could reflect the distinct functions played by BaiJ in the two strains.

Allo-bile acids are “flat” stereoisomers, in which the hydrogen at C5 is α-oriented instead of β-oriented. It is not completely clear what role allo-bile acids play for the host, but increased levels of some iso- and allo-forms of LCA have been observed in centenarians as compared to younger individuals and in colorectal cancer patients.^26–29^ We have not detected any allo-bile acids in experiments with *C. scindens* bacterial cultures, or in mice infected with a defined microbiota including *C. scindens* (SI Material and Methods).^21,30^ However, allo-DCA has been reported earlier in studies of 7α-dehydroxylation of CA by *C. scindens* VPI 12708.^31^ In a recent study, BaiJ from *C. scindens* strain ATCC 35704 and from strain VPI 12708 were shown to have 5α-reductase activity and responsible for reducing 3-oxo-Δ^4^-LCA (and 3-oxo-Δ^4^-DCA) to 3-oxo-allo-LCA (and 3-oxo-allo-DCA).^16^ We confirmed this finding (Figures 7b and 9a), and as discussed above, BaiJ from *C. scindens* ATCC 35704 is also responsible for the preceding reductive step during 7α-dehydroxylation with the five-enzyme set (Figure 6). Yet we found little, or no, 3-oxo-allo-LCA during the CDCA 7α-dehydroxylation experiments with the five-enzyme set containing BaiJ, with the majority of 7α-dehydroxylated bile acids being 3-oxoLCA (Figures 2b, 3, 5, 9b, S3, S5A). This result was quite surprising, given that BaiJ efficiently reduces 3-oxo-Δ^4^-LCA to 3-oxo-allo-LCA (approximately 24% of total bile acids after 3 hours, Figure 7b), whereas BaiCD reduces the same substrate to 3-oxo-LCA poorly (less than 1% of total bile acids after 24 hours, Figure 7a). One possible explanation for this discrepancy could be that the intermediate available for the second reductive step during 7α-dehydroxylation of CDCA with the five-enzyme set is not 3-oxo-Δ^4^-LCA, but rather 3-oxo-Δ^4^-LCA-CoA, and BaiJ and/or BaiCD reduce the CoA conjugate to 3-oxoLCA-CoA. We could not assess this directly because 3-oxo-Δ^4^-LCA-CoA is not commercially available, and we could not produce it because BaiB does not ligate CoA to 3-oxo-Δ^4^-LCA *in vitro* (data not shown). Instead, we opted to utilize the 3-oxo-Δ^4^-CDCA intermediate (because it is a substrate for CoA ligation by BaiB) and compared its further conversion by BaiE, BaiJ, and BaiCD with and without CoA conjugation (i.e., with and without BaiB) (Figure 8). We found that 3-oxoLCA was only produced when BaiB was present and was dependent on the presence of BaiCD, whereas 3-oxo-allo-LCA was most abundantly produced in the absence of the CoA-conjugating enzyme. This corroborates the co-existence of two branches of the pathway: one predominant in the absence of BaiB, and in which BaiJ reduces the unconjugated 3-oxo-Δ^4^-LCA intermediate to 3-oxo-allo-LCA; and a second in which BaiCD reduces the CoA-conjugated intermediate to produce 3-oxoLCA-CoA (Figure 11).

Interpretation of our various results leads us to believe that the vast majority of bile acids that proceed through 7-dehydroxylation are CoA-conjugated bile acids (Figure 11). Several lines of evidence support this assertion: (a) The first oxidative step by BaiA2, which produces 3-oxoCDCA from CDCA, can take place without CoA conjugation (Figure S7). But if BaiB is absent from the five-enzyme set, and therefore no CoA conjugation can take place, CDCA is converted almost exclusively to 3- and 7-oxidized bile acids (3-oxoCDCA, 7-oxoLCA and 3,7-dioxo-CDCA, Figure S5A) and never advances further, indicating that BaiCD has specificity for 3-oxoCDCA-CoA and not 3-oxoCDCA; (b) We could corroborate this by demonstrating that 3-oxoCDCA is converted to 3-oxo-Δ^4^-CDCA by BaiCD very poorly (less than 1% of total bile acids (Figure S5B) (c) In agreement with this, when the enzymes from the five-enzyme set were sequentially added to CDCA (Figure 5), we observed the formation of 3-oxo-Δ^4^-CDCA-CoA but not 3-oxo-Δ^4^-CDCA after BaiCD was supplied, strongly suggesting that BaiCD produces only 3-oxo-Δ^4^-CDCA-CoA; (d) Finally, in the same experiment, the substrate for the first reduction (3-oxo-Δ^4,6^-LCA) was only detected in CoA-ligated form prior to the addition of BaiJ and its subsequent reduction (Figure 5). Taken together, these data suggest that the majority of bile acids that proceed to the reductive arm of 7α-dehydroxylation from CDCA with the five-enzyme set are conjugated to CoA, and we hypothesize that this determines why minor amounts of allo-bile acids are produced (if any) (Figure 11).

This raises the question of when CoA is added and lost during the transformation of CDCA to LCA. In the six-enzyme 7α-dehydroxylation pathway, CoA is removed by the CoA transferase BaiF immediately before the reductive arm (Figure 11).^9^ This may be necessary for the reaction to proceed, as we observed that BaiF is needed if BaiH is present and BaiJ absent (Figure 2b). However, in the five-enzyme set, there is no BaiF and 7α-dehydroxylation still occurs. In that case, few non CoA-conjugated intermediates are observed and the intermediates in which the ring is oxidized are missing entirely (only 3-oxoCDCA, 3-oxoLCA, and LCA, and sometimes 3-oxo-allo-LCA are observed). In contrast, we observe almost all CoA-conjugated intermediates, including LCA-CoA, indicating that CoA may be ligated to both CDCA and 3-oxoCDCA, and then carried along to the conclusion of the pathway. How CoA is removed without BaiF remains to be established, but this reaction could be catalyzed by BaiB.

Recent work has established that there are variations in the organization of the *bai* operon and the genes it contains among microorganisms, but relatively little is known about *bai* genes external to the operon.^16,18,25,32–34^ This work demonstrates on the one hand that an enzyme encoded by a gene outside the *bai* operon (i.e., BaiJ) is necessary for efficient 7α-dehydroxylation at least in one *C. scindens* strain and, on the other, that enzymes from the operon can be replaced by others. This demonstrates that even within a single organism, there is redundancy in the enzymatic bile acid 7α-dehydroxylation pathway (Figure 11).

The CDCA 7α-dehydroxylation pathway discussed here for *C. scindens* strain ATCC 35704 differs from the one evidenced for CA in strain VPI 12708 in several ways (Figure 11). First, CoA is ligated either at CDCA or 3-oxoCDCA and released at the end of the pathway (LCA). Second, BaiJ, rather than BaiH, is the reductase for the first reductive step. Third, we postulate that the presence of CoA determines the outcome of the second reductive step. Fourth, BaiA2 and BaiA1/3 are interchangeable as the 3α-HSDH in this pathway. Finally, the last reductive step is reversible, suggesting that LCA efflux must occur rapidly to preclude its re-oxidation to 3-oxoLCA.

Further work is needed to determine the role of other proteins found in some organisms, such as BaiO, BaiN, BaiK, BaiL, and 12αHSDH, and establish whether they, such as BaiJ and BaiA1/3, can also replace *bai* operon enzymes in 7α-dehydroxylation of primary bile acids. This study used a simplified *in vitro* system to decipher a complex pathway, but it would be valuable to assess in future work how other bacterial species present in the gut microbiota, as well as the host factors, modulate or influence 7α-dehydroxylation *in vivo*.

## Material and methods

### Bacterial strains and culture conditions

Bacterial strains and plasmids used in this study are listed in Table S2.

*E. coli* strains were grown in LB medium or TB medium (for protein expression) at 37°C and supplemented with ampicillin (100 μg/mL), kanamycin (50 μg/mL), spectinomycin (50 μg/mL), or chloramphenicol (25 μg/mL) when indicated.

### Cloning of bai genes for protein expression

Genes encoding BaiA1/3, BaiA2, BaiCD, BaiH, BaiB, BaiF, BaiE, BaiJ, BaiO, BaiI, and BaiN from *Clostridium scindens* ATCC 35704 and BaiJ from *C. scindens* VPI 12708 (see Table S3 for gene loci and protein accession numbers) were amplified by PCR using primers listed in Table S4. Each PCR-amplified gene was cloned into pET28b+ or a modified pET28b+ vector (m-pET28b+) using Gibson Assembly (New England BioLabs), according to the manufacturer’s instructions. The modified pET28b+ carries both a His-Tag and a Strep-Tag sequence (K. Lau, unpublished). Restriction sites used for plasmid digestion are indicated in Table S4. The recombinant plasmids were transformed into *E. coli* TOP10 (One Shot™ TOP10 Chemically Competent *E. coli*, Invitrogen). All plasmid constructs were verified by sequencing and further transformed into the expression strain *E. coli* BL21-CodonPlus(DE3)-RIPL (Agilent).

### Protein expression and purification

Recombinant *E. coli* BL21-CodonPlus(DE3)-RIPL cells were grown on LB agar plates containing kanamycin, spectinomycin, and chloramphenicol. A single colony was used to start an overnight culture in 100 mL LB medium containing the antibiotics mentioned above. The overnight culture was used to inoculate 2 L of TB autoinduction medium. For the purification of BaiCD and BaiH, 600 μM cysteine and 200 μM FeCl_3_ were added to the medium. Cultures were maintained at 37°C under constant agitation for two hours until OD_600_ reached 0.5. Cultures were grown for approximately 18 hours at 18°C before being harvested by centrifugation at 15,000 × g. Cell pellets were stored at −20°C until purification.

In a typical purification, the cell pellet was resuspended in 30 mL lysis buffer (20 mM HEPES, pH 7.5, 300 mM NaCl) supplemented with 10% v/v glycerol, 5 μL Benzonase® nuclease (Merck), and one protease inhibitor cocktail tablet (Roche). For the purification of the flavoproteins (BaiJ, BaiO, BaiCD) 500 μM FAD and FMN were added to the cells prior to lysis. Cell lysis was performed using a French Press Emulsiflex-C3 (Avestin). The lysate was removed by centrifugation at 4°C for 40 min at 20,000 × g. Supernatant was filtered through a 0.45 μm filter and subsequently loaded onto a 5 mL HisTrap^TM^ excel column (GE Healthcare Life Sciences) or 5 mL Strep-Tactin® XT Superflow® column (Iba) for the purification of His-tagged protein and Strep-tagged proteins respectively, using an ÄTKA Purifier (GE Healthcare Life Sciences). The column was washed with 10 column volumes of lysis buffer before elution. The elution buffer for His-tagged proteins contained 500 mM NaCl, 10 mM HEPES (pH 7.5), and 500 mM imidazole (pH 7.5). The elution buffer for purification of Strep-tagged proteins contained 60 mM HEPES (pH 7.5), 30 mM Tris, 250 mM NaCl, and 50 mM biotin. Samples from the lysate, the column flow-through, and the eluted fractions were run on a NuPAGE^TM^ 4–12% Bis-Tris gel (Invitrogen) to verify the size of the purified protein and the purity of the eluted fractions. The fraction(s) containing the protein, based on absorbance at 280 nm, were pooled and dialyzed overnight in 2 L of dialysis buffer (20 mM HEPES pH 7.5, 150 mM NaCl). After dialysis, proteins were concentrated using Amicon Ultra-15 Centrifugal Filter Units (Merck). The final protein concentration was estimated using absorbance measurements at 280 nm, and the extinction coefficient calculated withe ExPASy ProtParam tool based on its amino acid sequence. Figure S1 shows the gels with the purified enzymes.

### *In vitro* enzyme assays

#### General setup of enzyme assay

Assays were performed in buffer (50 mM HEPES pH 7.5, 50 mM KCl, 0.5 mM tris(2-carboxyethyl)phosphine (TCEP)) containing a cofactor mix (500 μM of each NAD, NADP, NADH, NADPH, FMN, FAD, CoA, ATP, ADP) and 100 μM of chenodeoxycholic acid (CDCA) or other bile acid substrate. The buffer and enzymes (kept on ice) were purged under N_2_ flow for 1 hour. All subsequent steps were carried out in an anoxic chamber (Coy Laboratory Products, 95% N_2_, 5% H_2_) at 37°C.

Generally, for assays with a single enzyme, 10 μg of protein was used per reaction, and for assays with an enzyme mix, 10 μg of each enzyme was pooled together in a tube with buffer before purging. Reactions (100 μL) were initiated by the addition of a single enzyme or enzyme mix to the buffer-substrate solution and incubated at 37°C. Samples (20 μL) were collected and mixed with 50% v/v of methanol/H_2_O (180 μL) at times indicated to stop the reaction and kept at −20°C until analysis. All assays, as well as no-enzyme controls (NEC), were performed in triplicate. Bile acids were analyzed by LC/MS.

#### Sequential addition of enzymes

The reaction was initiated by adding 20 μg BaiB to 100 μM CDCA in buffer containing cofactor mix (final volume 200 μL). After 2.5 hours, 20 μL was sampled and 20 μg of BaiA2 (5 μL) was added to the assay. After an additional 1.5 hours, 20 μg of BaiCD, then 20 μg of BaiE, and finally 20 μg of BaiJ were added with sampling before each enzyme addition. Bile acids were analyzed by LC/MS.

#### Role of CoA in 3-oxo-Δ^4^-CDCA conversion

Individual assays with 3-oxo-Δ^4^-CDCA in buffer containing only CoA and ATP were incubated overnight (18 hours) with either 20 μg BaiB or an equal volume of buffer (in final volume of 50 μL), at which point samples were removed (10 μL) (Part A, Figure 8). Then, buffer containing cofactors were added, as well as additional enzymes to assays previously incubated with BaiB (BaiB-BaiE-BaiJ or BaiB-BaiE-BaiJ-BaiCD) or previously incubated only with buffer (BaiE-BaiJ or BaiE-BaiJ-BaiCD). The final concentration of all co-factors was 500 μM (except CoA and ATP; 1 mM) in a final volume of 100 μL. Samples (20 μL) were removed after 7 hours (Part B, Figure 8). Bile acids were analyzed by LC/MS.

### Bile acid standards

Bile acid standards were purchased from Steraloids or Toronto Research Chemicals and were used for the identification and quantification of bile acid samples. Information regarding the quantified bile acids is provided in Table S1. Authentic standards were used for all bile acids listed in Table S1. Extracted ion chromatograms (EICs) of all standards and experimentally observed compounds are shown in Figure S8.

CoA conjugates of CDCA, 3-oxoCDCA, 3-oxo-Δ^4^-CDCA, 7-oxoLCA, and 3,7-dioxoCDCA were synthesized by incubating 10 μg of BaiB in buffer (50 mM HEPES pH 7.5, 50 mM KCl, 0.5 mM TCEP) containing co-factor mix (500 μM of each NAD, NADP, NADH, NADPH, FMN, FAD, CoA, ATP, ADP) with 100 μM of bile acid substrate under anoxic conditions for 24 hrs. We were unable to synthesize CoA conjugates of LCA, 3-oxoLCA, 3-oxo-Δ^4^-LCA, and 3-oxo-Δ^4,6^-LCA and identification of these were based on mass. EICs of bile acid CoA conjugates that were enzymatically synthesized are shown in Figure S9.

### *In vitro* enzyme assay sample preparation

100 μL from each sample, which was previously diluted and 100 μL from each calibration standard, were transferred into individual wells of 2 mL 96-well plate. 50 μL of an ISTD solution (CA-d_4_, CDCA-d_4_, DCA-d_4_ and LCA-d_4_, each at 2 μM in methanol) was pipetted into each well. Immediately after the addition of ISTD, 600 μL of 0.2% formic acid in H_2_O was added to each sample or calibration standard level. The 96-well plate was shaken using an orbital shaker at 300 rpm and centrifuged at 3500 rpm for 5 min at 4°C.

The contents of the 96-well plate were extracted by solid-phase extraction using an Oasis HLB 96-well μElution plate. The extracted samples were dried in a Biotage® SPE Dry 96 at 20°C and reconstituted with 100 μL of MeOH/H_2_O (50/50). The plate was shaken using an orbital shaker at 300 rpm for 5 min and centrifuged at 3500 rpm for 5 min at 4°C. The samples were injected on the LC-HRMS system.

### Bile acid extraction from *E. coli* cells

Samples (1 mL) were vacuum-dried overnight (evaporated in a SpeedVac, RVC 2–33 CDplus Infrarot, Martin Christ, Germany). The dried cells were collected, and approximately 450 mg of 0.5 mm zirconium beads were added to each tube. 1200 μL of MeOH/H_2_O (2/1) + 0.1% formic acid was used as extraction solvent. Samples were homogenized in a Precellys 24 Tissue Homogenizer (Bertin Instruments, Montigny-le-Bretonneux, France) at 6500 rpm with a 2 × 20” beat and 20” rest. Homogenized samples were centrifuged at 21’000 rcf for 15 min at 4°C. 10 μL of each supernatant and 100 μL of each calibration standard were transferred into individual wells of a 2 mL 96-well plate. 50 μL of an ISTD solution (CA-d_4_, CDCA-d_4_, DCA-d_4_ and LCA-d_4_, each at 2 μM in methanol) was pipetted into each well. Immediately after the addition of ISTD, 600 μL of 0.2% formic acid in H_2_O was added to each sample or calibration standard level. The 96-well plate was shaken using an orbital shaker at 300 rpm and centrifuged at 3500 rpm for 5 min at 4°C.

The contents of the 96-well plate were extracted by solid-phase extraction using an Oasis HLB 96-well μElution plate. The extracted samples were dried in Biotage® SPE Dry 96 at 20°C and reconstituted with 100 μL of MeOH/H_2_O (50/50). The plate was shaken using an orbital shaker at 300 rpm for 5 min and centrifuged at 3500 rpm for 5 min at 4°C. The samples were injected on the LC-HRMS system.

### LC/MS analysis

The quantitative method was performed on an Agilent Ultra-high-performance liquid chromatography 1290 series coupled to an Agilent 6530 Accurate-Mass Q-TOF mass spectrometer. The separation was done on a Zorbax Eclipse Plus C18 column (2.1 × 100 mm, 1.8 μm) mounted with its guard column Zorbax Eclipse Plus C18 (2.1 × 5 mm, 1.8 μm), both provided by Agilent Technologies (Santa Clara, CA, USA). The column compartment was kept heated at 50°C. Two different solutions were used as eluents: ammonium acetate [5 mM] in water as mobile phase A, and pure acetonitrile as mobile phase B. A constant flow of 0.4 mL/min was maintained over 26 minutes of run time with the following gradient (expressed in eluent B percentage): 0–5.5 min, constant 21.5% B; 5.5–6min, 21.5–24.5% B; 6–10 min, 24.5–25% B; 10–10.5min, 25–29% B; 10.5–14.5 min, isocratic 29% B; 14.5–15 min, 29–40% B; 15–18 min, 40–45% B; 18–20.5 min, 45–95% B; 20.5–23 min, constant 95% B; 23–23.1 min, 95–21.5% B; 23.10–26 min, isocratic 21.50% B. The system equilibration was implemented at the end of the gradient for 3 minutes in initial conditions. The autosampler temperature was maintained at 10°C and the injection volume was 5 μL. Ionization mode was operated in negative mode for the detection using the Dual AJS Jet Stream ESI Assembly. The QTOF acquisition settings were configured in 4 GHz high-resolution mode (resolution 17,000 FWHM at m/z 1000), data storage in profile mode, and the high-resolution full MS chromatograms were acquired over the range of m/z 100–1700 at a rate of 3 spectra/s. The mass spectrometer was calibrated using ESI-L solution from Agilent Technologies every 6 hours to maintain the best mass accuracy. Source parameters were set as follows: drying gas flow, 8 L/min; gas temperature, 300°C; nebulizer pressure, 35 psi; capillary voltage, 3500 V; nozzle voltage, 1000 V. Data were processed using the MassHunter qualitative and quantitative software. In the quantitative method, 11 bile acids (Table S1) were quantified using external calibration curves. Calibration curves were made from 11 dilutions with concentrations ranging from 50 nM to 15,000 nM. Estimated detection limits were 50–100 nM. The quantification was corrected by the addition of internal standards (CA-d_4_, CDCA-d_4_, DCA-d_4_ and LCA-d_4_, each at 1 μM in methanol) to all samples and standards. Extracted ion chromatograms were generated using a retention time window of ±1 min and a mass extraction window of ±40 ppm around the theoretical mass of the targeted bile acid. Reproducibility between assays was assessed by determining the variation between the no-enzyme control (NEC) values obtained in assays with CDCA as the substrate. Values were all within 87–116% of the average concentration obtained (Table S5). Furthermore, the stability of the instruments was verified by determining the mass of the three internal standards in all samples during a single run (Figure S10). Most were within 6 ppm of the expected mass.

### Identification of CoA conjugates

The same acquisition method described in the previous paragraph was used to detect CoA-conjugated bile acids. However, the positive mode was chosen for better detection of these compounds. The retention time and the exact masses in positive mode (M+H+) of CDCA-CoA, 3-oxo-CDCA-CoA, 3-oxo-Δ^4^-CDCA-CoA, 3,7-dioxo-CDCA-CoA, and 7-oxo-LCA-CoA were defined based on the final products of our enzymatically synthesized standards (Figure S9). 3-oxo-LCA, LCA-CoA, 3-oxo-Δ^4,6^-LCA-CoA and 3-oxo-Δ^4^-LCA-CoA could not be produced this way and were difficult to separate by LC. These compounds were identified based only on their theoretical masses (M+H+). The final samples of the experiments were directly injected on the LC-HRMS without any treatment to avoid the loss of any CoA-conjugated bile acid.

### Heterologous expression of *bai* genes in *Escherichia coli*

#### Plasmid constructions

A miniTn7 delivery vector, pUC18R6KT-miniTn7T-Km,^35^ was used to insert genes from the *bai* operon into the genome of *E. coli* Mt1B1.^36^ The regions encoding *baiB-CD-E* or *baiB-CD-E-A2* were amplified by PCR with primers pUCmini-baiB_F and either pUCmini-baiE_R or pUCmini-baiA2_R (see Table S4 for primers) using genomic DNA from *C. scindens* ATCC 35704 as template. The *fnrS* promoter (*fnrS*_*P*_) from *E. coli* MG1655 was amplified with primers pUCmini(E)-fnrSp_F (for *baiB-A2*) or pUCmini(K)-fnrSp_F (for *baiB-*E) and pUCmini-fnrSp_R (for both). *fnrS*_*P*_ and *bai* genes were inserted into KpnI-EcoRI (for *baiB-E*) or EcoRI-SacI (for *baiB-A2*) digested pUC18R6KT-miniTn7T-Km using NEB Builder HiFi Assembly mix (New England Biolabs) to construct pUC18-miniTn7-baiB-E and pUC18-miniTn7-baiB-A2, respectively.

The *baiJ* gene from *C. scindens* ATCC 35704 was amplified with primers pKIKO-baiJ_F and R, and inserted into pKIKO-lacZ-Cm^37^ (digested with EcoRI and BamHI) together with the *fnrS* promoter (amplified with primers pKIKO-fnrSp_F and R) using the NEB Builder HiFi Assembly mix to construct pKIKO-baiJ.

pBAD-baiG was prepared by amplifying *baiG* (primers pBAD-baiG_F and R) and inserting the PCR product into EcoRI-SmaI-digested pBAD using the NEB Builder HiFi Assembly.

All plasmid constructs were verified by Sanger sequencing.

#### *Insertion of* baiB-E *and* baiB-A2 *into* attTn7 *site in chromosome of* E. coli *Mt1B1*

Insertion of *fnrS*_P_-*baiB-E* and *fnrS*_P_-*baiB-A2* into the *attTn7* locus in *E. coli* Mt1B1 was performed as previously described.^35^ Briefly, the miniTn7 delivery plasmid was co-electroporated with pTNS2^35^ (expressing the transposase) into *E. coli* Mt1B1. Clones were selected on LB agar plates with kanamycin, single colonies were streaked, insertion was verified by PCR (primers neoF and glmS_down), and loss of helper plasmid was confirmed. To remove the kanamycin resistance cassette, the pFLP3^35^ helper plasmid (expressing FLP recombinase) was introduced. Colonies were tested for absence of the kanamycin cassette and then grown on LB agar plates containing sucrose to promote loss of the helper plasmid. Single purified colonies without the kanamycin cassette (and helper plasmid) were selected and insertion verified by PCR using genome-specific primers (glms_down and DBZ-03745_R), followed by sequencing of the PCR product to confirm the presence of all inserted genes.

#### *Insertion of* baiJ *into lacZ gene in Mt1B1*

A fragment containing *fnrS*_P_-*baiJ* and the chloramphenicol resistance cassette flanked by *lacZ* homology arms was amplified by PCR using the primers lacZ-insert_F and lacZ-insert-R from pKIKO-baiJ.^37^ The DNA fragment was electroporated into the recipient strain containing plasmid pSIJ8^38^ that had been pre-induced with 15 mM arabinose for 45 min to express the lambda Red recombineering genes from the plasmid before processed for electroporation. Clones with insertions were selected by plating on LB agar plates supplemented with chloramphenicol and ampicillin and incubated at 30°C. Insertion of the fragment into the *lacZ* gene was confirmed by PCR (primers Cm-test and lacZ_RO). Next, the chloramphenicol resistance gene was removed by inducing the expression of the FLP recombinase from pSIJ8 (with 50 mM rhamnose for 4 hours), followed by growth on LB supplemented with ampicillin at 30°C. Single colonies were screened for absence of the chloramphenicol cassette and then grown at 37°C to induce loss of the temperature-sensitive plasmid pSIJ8. Clones cured of pSIJ8 were purified, the insertion of *baiJ* verified by PCR using genome-specific primers (lacZ_FO and RO), and the PCR product was sequenced to confirm the insertion of *fnrS*_*P*_*-baiJ*.

### CDCA transformation by *E. coli* expressing *bai* genes

Strains KM0c, KM02, KM03, and KM04 (Table S2, Figure S4) were grown aerobically in LB supplemented with ampicillin to exponential phase at 37°C and then transferred to an anoxic chamber. The cultures were diluted 100-fold in anoxic LB medium supplemented with ampicillin (100 μg/mL), glucose (20 mM), and arabinose (0.005%) and grown for an additional 2 hours. CDCA (200 μM) was added to 1.5 mL of the culture (in triplicate) and incubated at 37°C overnight in an anoxic chamber. The samples (1 mL) were frozen before processing for bile acid extraction.

## Supplementary Material

SI_all_revised_final clean.docx

## Data Availability

The data used in this manuscript are available at DOI 10.5281/zenodo.8263047

## References

[1] Hamilton JP, Xie G, Raufman JP, Hogan S, Griffin TL, Packard CA, Chatfield DA, Hagey LR, Steinbach JH, Hofmann AF. Human cecal bile acids: concentration and spectrum. Am J Physiol-Gastrointest Liver Physiol. 2007;293(1):G256–23. doi:10.1152/ajpgi.00027.2007. 17412828

[2] Ridlon JM, Kang D-J, Hylemon PB. Bile salt biotransformations by human intestinal bacteria. J Lipid Res. 2006;47(2):241–259. doi:10.1194/jlr.R500013-JLR200. 16299351

[3] Hofmann AF. The enterohepatic circulation of bile acids in mammals: form and functions. *Front Biosci*. 2009;Volume(14):2584. doi:10.2741/3399. 19273221

[4] Wahlström A, Sayin SI, Marschall H-U, Bäckhed F. Intestinal crosstalk between bile acids and microbiota and its impact on host metabolism. Cell Metab. 2016;24(1):41–50. doi:10.1016/j.cmet.2016.05.005. 27320064

[5] Lin S, Wang S, Wang P, Tang C, Wang Z, Chen L, Luo G, Chen H, Liu Y, Feng B. et al. Bile acids and their receptors in regulation of gut health and diseases. Prog Lipid Res. 2023;89:101210. doi:10.1016/j.plipres.2022.101210. 36577494

[6] Mallonee DH, White WB, Hylemon PB. Cloning and sequencing of a bile acid-inducible operon from eubacterium sp. strain VPI 12708. J Bacteriol. 1990;172(12):7011–7019. doi:10.1128/jb.172.12.7011-7019.1990. 2254270 PMC210822

[7] Ridlon JM, Devendran S, Alves JM, Doden H, Wolf PG, Pereira GV, Ly L, Volland A, Takei H, Nittono H. et al. The ‘*in vivo* lifestyle’ of bile acid 7α-dehydroxylating bacteria: comparative genomics, metatranscriptomic, and bile acid metabolomics analysis of a defined microbial community in gnotobiotic mice. Gut Microbes. 2020;11(3):381–404. doi:10.1080/19490976.2019.1618173. 31177942 PMC7524365

[8] Ridlon JM, Harris SC, Bhowmik S, Kang D-J, Hylemon PB. Consequences of bile salt biotransformations by intestinal bacteria. Gut Microbes. 2016;7(1):22–39. doi:10.1080/19490976.2015.1127483. 26939849 PMC4856454

[9] Funabashi M, Grove TL, Wang M, Varma Y, McFadden ME, Brown LC, Guo C, Higginbottom S, Almo SC, Fischbach MA. A metabolic pathway for bile acid dehydroxylation by the gut microbiome. Nature. 2020;582(7813):566–570. doi:10.1038/s41586-020-2396-4. 32555455 PMC7319900

[10] Mallonee DH, Hylemon PB. Sequencing and expression of a gene encoding a bile acid transporter from *Eubacterium* sp. strain VPI 12708. J Bacteriol. 1996;178(24):7053–7058. doi:10.1128/jb.178.24.7053-7058.1996. 8955384 PMC178615

[11] Mallonee DH, Adams JL, Hylemon PB. The bile acid-inducible *baiB* gene from *Eubacterium* sp. strain VPI 12708 encodes a bile acid-coenzyme A ligase. J Bacteriol. 1992;174(7):2065–2071. doi:10.1128/jb.174.7.2065-2071.1992. 1551828 PMC205821

[12] Bhowmik S, Jones DH, Chiu H-P, Park I-H, Chiu H-J, Axelrod HL, Farr CL, Tien HJ, Agarwalla S, Lesley SA. Structural and functional characterization of BaiA, an enzyme involved in secondary bile acid synthesis in human gut microbe. Proteins Struct Funct Bioinforma. 2014;82(2):216–229. doi:10.1002/prot.24353. PMC3992121 23836456

[13] Kang DJ, Ridlon, JM, Moore, DR, Barnes, S, Hylemon, PB. Clostridium scindens *baiCD* and *baiH* genes encode stereo-specific 7α/7β-hydroxy-3-oxo-Δ^4^-cholenoic acid oxidoreductases☆. Biochim Biophys Acta BBA - Mol Cell Biol Lipids. 2008;1781(1–2):16–25. doi:10.1016/j.bbalip.2007.10.008. PMC2275164 18047844

[14] Bhowmik S, Chiu H-P, Jones DH, Chiu HJ, Miller MD, Xu Q, Farr CL, Ridlon JM, Wells JE, Elsliger MA. et al. Structure and functional characterization of a bile acid 7α dehydratase B ai E in secondary bile acid synthesis. Proteins Struct Funct Bioinforma. 2016;84(3):316–331. doi:10.1002/prot.24971. PMC4755848 26650892

[15] Ridlon JM, Hylemon PB. Identification and characterization of two bile acid coenzyme a transferases from *Clostridium scindens*, a bile acid 7α-dehydroxylating intestinal bacterium. J Lipid Res. 2012;53(1):66–76. doi:10.1194/jlr.M020313. 22021638 PMC3243482

[16] Lee JW, Cowley ES, Wolf PG, Doden HL, Murai T, Caicedo KYO, Ly LK, Sun F, Takei H, Nittono H. et al. Formation of secondary allo-bile acids by novel enzymes from gut Firmicutes. Gut Microbes. 2022;14(1):2132903. doi:10.1080/19490976.2022.2132903. 36343662 PMC9645264

[17] Harris SC, Devendran S, Alves JMP, Mythen SM, Hylemon PB, Ridlon JM. Identification of a gene encoding a flavoprotein involved in bile acid metabolism by the human gut bacterium *Clostridium scindens* ATCC 35704. Biochim Biophys Acta BBA - Mol Cell Biol Lipids. 2018;1863(3):276–283. doi:10.1016/j.bbalip.2017.12.001. 29217478

[18] Heinken A, Ravcheev DA, Baldini F, Heirendt L, Fleming RMT, Thiele I. Systematic assessment of secondary bile acid metabolism in gut microbes reveals distinct metabolic capabilities in inflammatory bowel disease. Microbiome. 2019;7(1):75. doi:10.1186/s40168-019-0689-3. 31092280 PMC6521386

[19] MORRIS GN, WINTER J, CATO EP, RITCHIE AE, BOKKENHEUSER VD. *Clostridium scindens* sp. nov., a human intestinal bacterium with desmolytic activity on corticoids. Int J Syst Evol Microbiol. 1985;35(4):478–481. doi:10.1099/00207713-35-4-478.

[20] Marion S, Studer N, Desharnais L, Menin L, Escrig S, Meibom A, Hapfelmeier S, Bernier-Latmani R. *In vitro* and *in vivo* characterization of *Clostridium scindens* bile acid transformations. Gut Microbes. 2019;10(4):481–503. doi:10.1080/19490976.2018.1549420. 30589376 PMC6748637

[21] Vico-Oton E, Volet C, Jacquemin N, Dong Y, Hapfelmeier S, Meibom KL, Bernier-Latmani R. Strain-Dependent Induction Of Primary Bile Acid 7-Dehydroxylation By Cholic Acid. 2022. doi:10.1101/2022.02.15.480494. PMC11293179 39090543

[22] Yoshimoto T, Higashi H, Kanatani A, Lin XS, Nagai H, Oyama H, Kurazono K, Tsuru D. Cloning and sequencing of the 7 alpha-hydroxysteroid dehydrogenase gene from *Escherichia coli* HB101 and characterization of the expressed enzyme. J Bacteriol. 1991;173(7):2173–2179. doi:10.1128/jb.173.7.2173-2179.1991. 2007545 PMC207764

[23] Devendran S, Shrestha R, Alves JMP, Wolf PG, Ly L, Hernandez AG, Méndez-García C, Inboden A, Wiley J, Paul O. et al. *Clostridium scindens* ATCC 35704: integration of nutritional requirements, the complete genome sequence, and global transcriptional responses to bile acids. Appl Environ Microbiol. 2019;85(7):e00052–19. doi:10.1128/AEM.00052-19. 30737348 PMC6585500

[24] Mallonee DH, Lijewski MA, Hylemon PB. Expression in *Escherichia coli* and characterization of a bile acid-inducible 3alpha-hydroxysteroid dehydrogenase from *Eubacterium* sp. strain VPI 12708. Curr Microbiol. 1995;30(5):259–263. doi:10.1007/BF00295498. 7766153

[25] Ridlon JM, Kang D-J, Hylemon PB. Isolation and characterization of a bile acid inducible 7α-dehydroxylating operon in *Clostridium hylemonae* TN271. Anaerobe. 2010;16(2):137–146. doi:10.1016/j.anaerobe.2009.05.004. 19464381 PMC6262846

[26] Tadano T, Kanoh M, Kondoh H, Matsumoto M, Mimura K, Kanoh Y, Sakamoto K, Kamano T. Kinetic analysis of bile acids in the feces of colorectal cancer patients by gas chromatography-mass spectrometry (GC-MS). Rinsho Byori. 2007;55:417–427. 17593686

[27] Tadano T, Kanoh M, Matsumoto M, Sakamoto K, Kamano T. Studies of serum and feces bile acids determination by gas chromatography-mass spectrometry. Rinsho Byori. 2006;54:103–110. 16548228

[28] Sato Y, Atarashi K, Plichta DR, Arai Y, Sasajima S, Kearney SM, Suda W, Takeshita K, Sasaki T, Okamoto S. et al. Novel bile acid biosynthetic pathways are enriched in the microbiome of centenarians. Nature. 2021;599(7885):458–464. doi:10.1038/s41586-021-03832-5. 34325466

[29] Shiffka SJ, Kane MA, Swaan PW. Planar bile acids in health and disease. Biochim Biophys Acta BBA - Biomembr. 2017;1859(11):2269–2276. doi:10.1016/j.bbamem.2017.08.019. PMC5734676 28887043

[30] Marion S, Desharnais L, Studer N, Dong Y, Notter MD, Poudel S, Menin L, Janowczyk A, Hettich RL, Hapfelmeier S. et al. Biogeography of microbial bile acid transformations along the murine gut. J Lipid Res. 2020;61(11):1450–1463. doi:10.1194/jlr.RA120001021. 32661017 PMC7604727

[31] Hylemon P, Melone P, Franklund C, Lund E, Björkhem I. Mechanism of intestinal 7 alpha-dehydroxylation of cholic acid: evidence that allo-deoxycholic acid is an inducible side-product. J Lipid Res. 1991;32(1):89–96. doi:10.1016/S0022-2275(20)42247-3. 2010697

[32] Jin W-B, Li T-T, Huo D, Qu S, Li XV, Arifuzzaman M, Lima SF, Shi H-Q, Wang A, Putzel GG. et al. Genetic manipulation of gut microbes enables single-gene interrogation in a complex microbiome. Cell. 2022;185(3):547–562.e22. doi:10.1016/j.cell.2021.12.035. 35051369 PMC8919858

[33] Bai Y, Zhao T, Gao M, Zou Y, Lei X. A novel gene alignment in *Dorea* sp. AM58-8 produces 7-dehydroxy-3β bile acids from primary bile acids. Biochem. 2022;61(24):2870–2878. doi:10.1021/acs.biochem.2c00264. 36130198

[34] Song I, Gotoh Y, Ogura Y, Hayashi T, Fukiya S, Yokota A. Comparative genomic and physiological analysis against *Clostridium scindens* reveals *Eubacterium* sp. c-25 as an atypical deoxycholic acid producer of the human gut microbiota. Microorgan. 2021;9(11):2254. doi:10.3390/microorganisms9112254. PMC8623032 34835380

[35] Choi K-H, Gaynor JB, White KG, Lopez C, Bosio CM, Karkhoff-Schweizer RR, Schweizer HP. A Tn7-based broad-range bacterial cloning and expression system. Nat Methods. 2005;2(6):443–448. doi:10.1038/nmeth765. 15908923

[36] Lagkouvardos I, Pukall R, Abt B, Foesel BU, Meier-Kolthoff JP, Kumar N, Bresciani A, Martínez I, Just S, Ziegler C. et al. The mouse intestinal bacterial collection (miBC) provides host-specific insight into cultured diversity and functional potential of the gut microbiota. Nat Microbiol. 2016;1(10):1–15. doi:10.1038/nmicrobiol.2016.131. 27670113

[37] Sabri S, Steen JA, Bongers M, Nielsen LK, Vickers CE. Knock-in/Knock-out (KIKO) vectors for rapid integration of large DNA sequences, including whole metabolic pathways, onto the *Escherichia coli* chromosome at well-characterised loci. *Microb Cell Fact*. 2013;12(1):60. doi:10.1186/1475-2859-12-60. 23799955 PMC3706339

[38] Jensen SI, Lennen RM, Herrgård MJ, Nielsen AT. Seven gene deletions in seven days: fast generation of *Escherichia coli* strains tolerant to acetate and osmotic stress. Sci Rep. 2015;5(1):17874. doi:10.1038/srep17874. 26643270 PMC4672327

