# Supplementary material for: BaiJ and BaiB are key enzymes in the chenodeoxycholic acid 7α-dehydroxylation pathway in the gut microbe Clostridium scindens ATCC 35704
Source: Gut Microbes. 2024 Mar 11;16(1):2323233. doi: 10.1080/19490976.2024.2323233 (PMC10936602; doi:10.1080/19490976.2024.2323233)
Supplement: SI_all_revised_final clean.docx [file KGMI_A_2323233_SM0277.docx]

**Supplementary information**

**BaiJ and BaiB are key enzymes in the chenodeoxycholic acid 7**α**-dehydroxylation pathway in the gut microbe *Clostridium scindens* ATCC 35704**

Karin Lederballe Meibom^1†^***, Solenne Marion^1^***, Colin Volet^1^, Théo Nass^1^, Eduard Vico-Oton^1^, Laure Menin^2^, Rizlan Bernier-Latmani^1†^

Author information:

*^1^Environmental Microbiology Laboratory, École Polytechnique Fédérale de Lausanne (EPFL), Lausanne, Switzerland*

*^2^Institute of Chemical Sciences and Engineering, École Polytechnique Fédérale de Lausanne (EPFL), Lausanne, Switzerland*

** These authors contributed equally to the work*

^†^Corresponding authors:

Karin Lederballe Meibom

Email: karin.meibom@epfl.ch

Rizlan Bernier-Latmani

Email: rizlan.bernier-latmani@epfl.ch

**Material and Methods**

***Identification of allo-bile acids in bacterial and mice samples***

Samples from a stable gnotobiotic murine model, Oligo-Mouse-Microbiota (Oligo-MM12) supplemented with *C. scindens* ATCC 35704 (sDMDMm2 + *C. scindens*)^1^ and of *C. scindens* ATCC 35704 grown *in vitro* in the presence of CDCA^2^, previously not assessed for the presence of allo-bile acids, were re-analyzed. No allo-LCA or 3-oxo-allo-LCA was detected in any sample.

Table S1. List of bile acids quantified with the LC-MS.

| **Common name** | **Abbreviation** | **Systematic name^a^** | **Structure** | **m/z^b^** |
| --- | --- | --- | --- | --- |
| Chenodeoxycholic acid | CDCA | 3α,7α-dihydroxy-5β-cholan-24-oic acid |  | 391.2854 |
| 3-oxochenodeoxycholic acid | 3-oxoCDCA | 7α-hydroxy-3-oxo-5β-cholan-24-oic acid |  | 389.2697 |
| Lithocholic acid | LCA | 3α-hydroxy-5β-cholan-24-oic acid |  | 375.2905 |
| Allolithocholic acid | allo-LCA | 3α-hydroxy-5α-cholan-24-oic acid |  | 375.2905 |
| 3-oxolithocholic acid | 3-oxoLCA | 3-oxo-5β-cholan-24-oic acid |  | 373.2748 |
| 3-oxo-allolithocholic acid | 3-oxo-allo-LCA | 3-oxo-5α-cholan-24-oic acid |  | 373.2748 |
| 7-oxolithocholic acid | 7-oxoLCA | 3α-hydroxy-7-oxo-5β-cholan-24-oic acid |  | 389.2697 |
| 3,7-dioxochenodeoxycholic acid | 3,7-dioxoCDCA | 3,7-dioxo-5β-cholan-24-oic acid |  | 387.2541 |
| 3-oxo-delta4-chenodeoxycholic acid | 3-oxo-Δ^4^-CDCA | 7a-hydroxy-3-oxochol-4-en-24-oic acid |  | 387.2541 |
| 3-oxo-delta4,6-lithocholic acid | 3-oxo-Δ^4,6^-LCA | 3-oxochola-4,6-dien-24-oic Acid |  | 369.2435 |
| 3-oxo-delta4-lithocholic acid | 3-oxo-Δ^4^-LCA | 3-oxochol-4-en-24-oic acid |  | 371.2592 |

^a^ according to LIPID MAPS

^b^ in negative mode

For experiments in Figures 2A, 6, and S6 we did not quantify 3-oxo-allo-LCA and allo-LCA as no such forms are relevant; BaiJ was not included in the assay in data for Figure 2A and allo-forms are not relevant for the specific reactions shown in Figure 6 (first reductive step) and Figure S6 (third reductive step from 3-oxoLCA). The minor amount listed as 3-oxoLCA in the data file for Figure 6 (see data file Fig 6_quantification in data repository, https://doi.org/10.5281/zenodo.8263047) is likely 3-oxo-allo-LCA (and not shown in graph) formed as BaiJ can catalyze the formation of 3-oxo-allo-LCA in the second reductive step.

**Table S2. Bacterial strains and plasmids used in the study.**

| **Strain or plasmid** | | **Description** | **Reference/source** |
| --- | --- | --- | --- |
|  | |  |  |
| *Clostridium scindens* | |  |  |
|  | ATCC 35704 | Isolated from human fecal sample | ^3^ |
|  | VPI 12708 | Isolated from human stool sample | ^4^ |
|  |  |  |  |
| *Escherichia coli* | |  |  |
|  | TOP10 | F- *mcrA* Δ(*mrr-hsd*RMS-*mcr*BC) Φ80*lac*ZΔM15 Δ *lac*X74 *rec*A1 *ara*D139 Δ( *araleu*)7697 *gal*U *gal*K *rps*L (Str^R^) *end*A1 *nup*G | Invitrogen |
|  | DH5αλ*pir* | F^-^ Φ80*lacZ*ΔM15 *endA1 recA1 hsdR17 supE44-thi-1 gyrA96 relA1* (*lacZYA-*argF)*U169*l λ*pir* lysogen | Lab collection |
|  | BL21-CodonPlus(DE3)-RIPL | *E. coli* B; F^-^ *ompT hsdS*(r_B_^-^ m_B_^-^) *dcm*^+^ Tet^r^ *gal* λ(DE3) *endA* Hte [argU proL Cam^r^] [*argU ileY leuW* Strep/Spec^r^] | Agilent |
|  | MG1655 | K-12 wild-type strain | Lab collection |
|  | Mt1B1 | Mouse isolate | DSMZ^5^ |
|  | KM0c | Mt1B1 + pBAD-baiG | This study |
|  | KM02 | Mt1B; *fnrS_p_-baiB-CD-E*; *fnrS_p_-baiJ* + pBAD-baiG | This study |
|  | KM03 | Mt1B1 *fnrS_p_-baiB-CD-E-A2* + pBAD-baiG | This study |
|  | KM04 | Mt1B1 *fnrS_p_-baiB-CD-E-A2*; *fnrS_p_-baiJ* + pBAD-baiG | This study |
|  |  |  |  |
| Plasmids | |  |  |
|  | pET28b+ | Expression vector, His-Tag, Km^r^ | Lab collection |
|  | m-pET28b+ | pET28b+ with additional Strep-Tag | K. Lau |
|  | pBAD-SmaI | Expression vector with arabinose inducible promoter, Amp^r^ | M. Blokesch |
|  | pUC18R6KT-miniTn7T-Km | MiniTn7 delivery plasmid, Km^r^, Amp^r^ | ^6^ |
|  | pTNS2 | Plasmid for transposase expression, Km^r^ | ^6^ |
|  | pFLP3 | Plasmid for flippase recombinase expression, Amp^r^,Tc^r^ | ^6^ |
|  | pKIKO-lacZCm | Plasmid for integration in *E. coli lacZ*, Cm^r^ | ^7^ |
|  | pSIJ8 | Temperature sensitive plasmid for either lambda Red recombinase genes or flippase recombinase expression, Amp^r^ | ^8^ |
|  | pET28-baiE | *baiE* gene from *C. scindens* ATCC 35704 cloned with His-Tag in pET28b+ | This study |
|  | pET28-baiN | *baiN* gene from *C. scindens* ATCC 35704 cloned with His-Tag in pET28b+ | This study |
|  | m-pET28-baiB | *baiB* gene from *C. scindens* ATCC 35704 cloned with Strep-Tag in m-pET28b+ | This study |
|  | m-pET28-baiCD | *baiCD* gene from *C. scindens* ATCC 35704 cloned with Strep-Tag in m-pET28b+ | This study |
|  | m-pET28-baiA2 | *baiA2* gene from *C. scindens* ATCC 35704 cloned with Strep-Tag in m-pET28b+ | This study |
|  | m-pET28-baiA1/3 | *baiA1/3* gene from *C. scindens* ATCC 35704 cloned with Strep-Tag in m-pET28b+ | This study |
|  | m-pET28-baiF | *baiF* gene from *C. scindens* ATCC 35704 cloned with Strep-Tag in m-pET28b+ | This study |
|  | m-pET28-baiH | *baiH* gene from *C. scindens* ATCC 35704 cloned with Strep-Tag in m-pET28b+ | This study |
|  | m-pET28-baiJ | *baiJ* gene from *C. scindens* ATCC 35704 cloned with Strep-Tag in m-pET28b+ | This study |
|  | m-pET28-baiI | *baiI* gene from *C. scindens* ATCC 35704 cloned with Strep-Tag in m-pET28b+ | This study |
|  | m-pET28-baiO | *baiO* gene from *C. scindens* ATCC 35704 cloned with Strep-Tag in m-pET28b+ | This study |
|  | m-pET28-baiJ-VPI | *baiJ* gene from *C. scindens* VPI 12708 cloned with Strep-Tag in m-pET28b+ | This study |
|  | pUC18-miniTn7-baiB-E | MiniTn7 delivery plasmid with *baiB-CD-E* from *C. scindens* ATCC expressed from *E. coli fnrS* promoter | This study |
|  | pUC18-miniTn7-baiB-A2 | MiniTn7 delivery plasmid with *baiB-CD-E-A2* from *C. scindens* ATCC expressed from *E. coli fnrS* promoter | This study |
|  | pKIKO-baiJ | Plasmid for integration of *baiJ* from *C. scindens* ATCC 35704 expressed from *E. coli fnrS* promoter into *lacZ* | This study |
|  | pBAD-baiG | *baiG* from *C. scindens* ATCC 35704 cloned under control of arabinose-inducible promoter in pBAD-SmaI | This study |
|  | |  |  |

**Table S3. Genes and protein IDs of expressed and purified proteins.**

| **Locus** | **Gene** | **Protein ID** |
| --- | --- | --- |
| HDCHBGLK_01430 | *baiB* | WP_009247906.1 |
| HDCHBGLK_01431 | *baiCD* | WP_004607864.1 |
| HDCHBGLK_01432 | *baiE* | WP_009247905.1 |
| HDCHBGLK_01433 | *baiA2* | WP_004607862.1 |
| HDCHBGLK_01434 | *baiF* | WP_004607861.1 |
| HDCHBGLK_01435 | *baiG* | WP_004607860.1 |
| HDCHBGLK_01436 | *baiH* | WP_004607859.1 |
| HDCHBGLK_01437 | *baiI* | WP_004607858.1 |
| HDCHBGLK_02470 | *baiA1/3* | WP_004607746.1 |
| HDCHBGLK_03018 | *baiN* | WP_004606074.1 |
| HDCHBGLK_03019 | *baiO* | WP_039909528.1 |
| HDCHBGLK_03451 | *baiJ* | WP_004605051.1 |
| GGADHKLB_RS07120 | *baiJ-VPI* | WP_025644103.1 |

**Table S4. Oligonucleotides used in the study.**

| **Name** | **Sequence (5'-3')** |
| --- | --- |
| Primers for cloning *bai* genes into protein expression vector (pET28b+ or m-pET28b+) | |
| baiA1/3_F^a^ | GCA GTT CGA AAA GGG ATC CGG ATC CAT GAA ACT TGT ACA GGA CAA A |
| baiA1/3_R^a^ | GGT GCT CGA GTG CGG CCG CCT ACT ATG GCC TGT AAG CCC CAT C |
| baiA2_F^a^ | GCA GTT CGA AAA GGG ATC CGG ATC CAT GAA TCT CGT ACA AGA CAA G |
| baiA2_R^a^ | GGT GCT CGA GTG CGG CCG CCT ATT ATG GTC TGT AAG CTC CGT C |
| baiCD_F^b^ | GCA GTT CGA AAA GGG ATC CAT GAG TTA CGA AGC ACT TTT T |
| baiCD_R^b^ | GGT GCT CGA GTG CGG CCG CCT ACT AGA TTG CCA TTC CTG C |
| baiH_F^b^ | GCA GTT CGA AAA GGG ATC CAT GGA TAT GAA ACA TTC CAG A |
| baiH_R^b^ | GGT GCT CGA GTG CGG CCG CCT ATT ACA GGC TGT ATG CCT T |
| baiB_F^a^ | GCA GTT CGA AAA GGG ATC CGG ATC CAT GCA CAA AAA ATC AAC GTG T |
| baiB_R^a^ | GGT GCT CGA GTG CGG CCG CCT ATC ATA CCC CGC GGG CAA TAC A |
| baiF_F^a^ | GCA GTT CGA AAA GGG ATC CGG ATC CAT GGC TGG ATT AAA AGA TTT T |
| baiF_R^a^ | GGT GCT CGA GTG CGG CCG CCT ATT ACT CCT CTT TCT TTC TCA T |
| baiE_F^c^ | GCC GCG CGG CAG CCA TAT GAT ACA TAT GAC ATT AGA AGA GAG |
| baiE_R^c^ | CCA GTC ATG CTA GCC ATT ATT TGT GCA TGT TCA TCG |
| baiI_F^b^ | GCA GTT CGA AAA GGG ATC CAT GGC AGT GAA GGC AAT CTC A |
| baiI_R^b^ | GCA GTT CGA AAA GGG ATC CGG ATC CAT GGC AAG TTA TAC ACC CGG G |
| baiJ_F^a^ | GCA GTT CGA AAA GGG ATC CGG ATC CAT GGC AAG TTA TAC ACC CGG G |
| baiJ_R^a^ | GGT GCT CGA GTG CGG CCG CCT ATT ACA GCA TCT CTC TCT G |
| baiO_F^a^ | GCA GTT CGA AAA GGG ATC CGG ATC CAT GAT ATC TAT GAT TCG GAT C |
| baiO_R^a^ | GGT GCT CGA GTG CGG CCG CCT ATT AAA AAT TTC TTA AGC CTT T |
| baiN_F^d^ | CTT TAA GAA GGA GAT ATA CCA TGA ATC GGA TTG GAA TCA TCG GAG G |
| baiN_R^d^ | TGG TGG TGG TGG TGC GCG GGT CTT TCC GCC GCC GC |
| baiJ-VPI_F^a^ | CCA CCC GCA GTT CGA AAA GGG ATC CGG ATC CAT GGC ACA TTA TGT TCC AG |
| baiJ-VPI_R^a^ | TGG TGG TGG TGC TCG AGT GCT TAA AGC ATT GAC TGT CC |
| Primers for cloning *bai* genes into pUC18R6KT-miniTn7T | |
| pUCmini(E)-fnrSp_F (A) | GGT ACC GGG CCC AAG CTT CTC GAG GAA AAA CGC CGC AAA GTT TG |
| pUCmini(K)-fnrSp_F (K) | GGC CTG CAA GGC CTT CGC GAG GTA CAA AAA CGC CGC AAA GTT TG |
| pUCmini-fnrSp_R (B) | ACA TTT TTT CAA AAG AGA GAT ATT GCC CTG |
| pUCmini-baiB_F (C) | CAA TAT CTC TCT TTT GAA AAA ATG TAC AAA TAG CAC |
| pUCmini-baiE_R (L) | ACT AGT GGA TCC CCC GGG CTG CAG GGG GCC CTT ATT TGT GCA TGT TCA TCG |
| pUCmini-baiA2_R (L2) | GCT AAT TCG AGA TCA TGC ATG AGC TTT ATG GTC TGT AAG CTC C |
| Primers for cloning *baiJ* into and amplifying fragment from pKIKO-lacZCm | |
| pKIKO-fnrSp_F | ATG ATT GCA TGC GGT ACC GAG CTC GAA AAA CGC CGC AAA GTT TG |
| pKIKO-fnrSp_R | TTC TTC ATC TCT AAG AGA GAT ATT GCC CTG |
| pKIKO-baiJ_F | CAA TAT CTC TCT TAG AGA TGA AGA AAG GAG AG |
| pKIKO-baiJ_R | TTG CGC GTC GAC TGC GCA CTG CAG GTT ACA GCA TCT CTC TCT G |
| lacZ-insert_F | ATT TCC CCG AAA AGT GCC AC |
| lacZ-insert_R | GAC ATG GGA ATT AGC CAT GG |
| Primers for cloning *baiG* into pBAD | |
| pBAD-baiG_F | GCT AAC AGG AGG AAT TAC CCA TGA GCA CCG TAG CCA ATC |
| pBAD-baiG_R | TTG TTC GGG CCC AAG CTT CGT TAT GCC TCT TTC TTC TGA TAG ATT C |
| Primers for checking insertions in *E. coli* genome | |
| glmS_down | GCA CAT TAT TGA GAT GCC GCA |
| DBZ-03745_R | CGG TTT CTT TCT GGT AAG TG |
| neoF | CGT TGG CTA CCC GTG ATA TT |
| lacZ_FO | GCC GTC TGA ATT TGA CCT G |
| lacZ_RO | GGT CAC TTC GAT GAT TCG C |
| Cm-test | GGA GTG AAT ACC ACG ACG AT |

^a^ cloned in BamHI-NotI digested m-pET28b+

^b^ cloned in NcoI-NotI digested m-pET28b+

^c^ cloned in NdeI digested pET28b+

^d^ cloned in NcoI-XhoI digested pET28b+

**Table S5. Reproducibility between assays using CDCA as a substrate.**

| **Figure^a^** | **CDCA concentration in μM^b^** | | |
| --- | --- | --- | --- |
|  | **NEC 1** | **NEC 2** | **NEC 3** |
| Figure 2A | 91.913 (114) | 87.755 (108) | 93.864 (116) |
| Figure 2B | 82.042 (101) | 84.587 (105) | 85.373 (105) |
| Figure 3 | 72.805 (90) | 70.774 (87) | 76.172 (94) |
| Figure 5 | 77.833 (96) | 76.535 (95) | 78.518 (97) |
| Figure 9B | 74.501 (92) | 74.538 (92) | 75.597 (93) |
| Figures S3 and S5 | 74.583 (92) | 78.006 (96) | 77.615 (96) |
| Figure S7 | 91.332 (113) | 86.457 (107) | 89.016 (110) |

^a^ data from the 3 replicates of the no-enzyme control (NEC) from experiment shown in indicated figure.

^b^ Concentration in each sample is shown in µM. In parentheses, the concentration is shown in % relative to the average value (80.944 μM).

**Figure S1. Purified proteins.** Gels showing the purity of the proteins after expression and purification from *E. coli* BL21-CodonPlus(DE3)-RIPL. The second band for BaiO (around 55 kDA) is believed to be due to degradation of the protein.

**Figure S2. Bile acid 7-dehydroxylation intermediates conjugated to CoA detected during CDCA time-course experiment with the five-enzyme set (BaiB, BaiCD, BaiE, BaiA2, BaiJ).** Quantified non-CoA bile acids are shown in Figure 3. BAs with * are only identified on the basis of their mass, whereas the others on the basis of mass and enzymatically produced standards. 3-oxo-Δ^4^-CDCA-CoA could not be identified in this assay as it was not well separated from the 3,7-dioxoCDCA-CoA. 7-oxoLCA-CoA was detected at all times but not included here. 3-oxoLCA-CoA could not be determined due to a technical problem.

**Figure S3. BaiA2 can be replaced by BaiA1/3 in the five-enzyme set.** The five-enzyme set, BaiB, BaiCD, BaiE, BaiJ, and either BaiA2 or BaiA1/3 (as indicated), was incubated with CDCA for 7 hours and bile acids quantified. No 3-oxo-Δ^4^-CDCA, 3-oxo-Δ^4,6^-LCA, 3-oxo-Δ^4^-LCA, or allo-LCA were detected. Only CDCA was detected for the no enzyme control (NEC). Histograms depict the mean and standard deviation of three assays. Incomplete mass balance is attributed to the formation of CoA conjugates that are not quantifiable due to the absence of standards. The structure of bile acids discussed here is listed in Table S1.

**Figure S4. *E. coli* Mt1B1 strains engineered to express various *bai* genes.** (A) All strains contain a plasmid expressing *baiG* from an arabinose-inducible promoter. Portions of the *bai* operon; *baiB-E* (KM02) or *baiB-A2* (KM03 and KM04) were inserted in the *att*Tn7 locus and expressed from the *fnrS* promoter that is known to be active under microaerophilic and anaerobic conditions^9,10^. *baiJ* was inserted into the *lacZ* gene (strains KM02 and KM04) and also expressed from the *fnrS* promoter. (B) All bile acids detected after incubation of engineered *E. coli* strains with 200 μM CDCA and sampled after 24 hours. The last bar corresponds to the five-enzyme set plus BaiG. The concentrations of selected bile acids from the same experiment are shown in Figure 4. The *bai* genes in each strain are shown below bars. Histograms depict the mean and standard deviation of three assays. The structure of all bile acids discussed here is listed in Table S1.

**Figure S5. CDCA 7-dehydroxylation proceeds poorly through the oxidative arm without CoA conjugation.** (A) CDCA incubated with the five-enzyme (BaiB, BaiCD, BaiE, BaiA2, BaiJ) or four-enzyme (BaiCD, BaiE, BaiA2, BaiJ) set for 7 hours and bile acids quantified. Minor amounts of 3-oxo-allo LCA were produced with and without BaiB (0.25 ± 0.04 μM and 0.34 ± 0.07 μM). Very little 3-oxoLCA was produced without BaiB (0.16 ± 0.07 μM) compared to with BaiB (11.70 ± 0.40 μM). (B) 3-oxoCDCA was incubated with either BaiCD or BaiJ for 3 hours and bile acids quantified. A minor amount of 3-oxo-Δ^4^-CDCA (1.09 ± 0.18 μM) was produced with BaiCD but not with BaiJ. Very small amounts (<0.15 μM) of some bile acids were detected in all samples (also NEC), but believed to be carry-over in the instrument and were not considered in the figure. Histograms depict the mean and standard deviation of three assays. The structure of bile acids discussed here is listed in Table S1.

**Figure S6. BaiA2 and BaiA1/3 re-oxidizes LCA.** BaiA2 or BaiA1/3 (as indicated) was incubated with LCA for 3 hours and bile acids quantified. Only LCA and 3-oxoLCA was observed. Histograms depict the mean and standard deviation of three assays.

**Figure S7. BaiJ and BaiCD oxidize the hydroxyl group at C7.** Individual enzymes (as indicated) were incubated with 100 μM CDCA for 3 hours and bile acids quantified. Only CDCA, 3-oxoCDCA, or 7-oxoLCA were observed. Histograms depict the mean and standard deviation of three assays. The slight oxidation of the hydroxyl group at C7 by BaiB, BaiE and BaiA2 may be due to a minor secondary activity of these enzymes or co-purification of another enzyme with this activity. Only CDCA was detected in the NEC. The structure of bile acids discussed here is listed in Table S1.

 ****

**Figure S8. Extracted ion chromatograms (EICs) of bile acids.** (A) Combined EICs from a mix of all standards used for external calibration curves. Each bile acid was identified based on mass and the retention time obtained from LC-MS analysis of the single bile acid. The minor peaks seen inside another peak are the m+2 of the corresponding bile acid. Individual peaks were smoothed and scaled to maximum to improve visualization. (B) For each bile acid we show the EIC of an experimentally observed compound and of the authentic standard (extracted most of the time from the standards mix during same LC-MS run). For CDCA and 3-oxo-Δ^4^-CDCA, the experimentally observed compound were substrates in the experiment, whereas the remaining bile acids were products in the assay. For bile acids for which two compounds with same mass exist (3-oxoCDCA/7-oxoLCA; 3-oxo-Δ^4^-CDCA/3,7-dioxoCDCA; 3-oxoLCA/3-oxo-allo-LCA; LCA/allo-LCA), both appear in the EIC presented of the authentic standard but are identified by retention time. For the two sets of chromatograms extracted for mass 387.2541 (3-oxo-Δ^4^-CDCA/3,7-dioxoCDCA), we observed a slight shift in retention time, possibly due to that the samples were not run at the same time. Individual peaks were smoothed. The retention time is indicated at the peak.

 ****

**Figure S9. Extracted ion chromatograms (EICs), acquired in positive mode, of enzymatically produced CoA-conjugated bile acids.** For each bile acid substrate, we show in the upper panel the no-enzyme control (to verify the measured m/z in positive mode) and in the lower panel the product after incubation with BaiB. Individual peaks were smoothed. In each case, a new compound was observed after BaiB incubation. Retention times are indicated at the specific peaks.

(A) The m/z of CDCA observed in positive mode differed from the calculated (measured m/z: 357.2736 vs. calculated m/z: 393.2999) but the retention time was conserved. The peak assigned to CDCA-CoA has the same measured m/z as the calculated (m/z: 1142.4046).

(B) 3-oxoCDCA in positive mode resulted in three peaks with same retention time but different masses (m/z: 355.2581, 373.2685, and 408.3054) formed through differences in the extent of protonation, none of which corresponds to the calculated (m/z: 391.2843). An overlay of the three peaks is shown in the figure. The CoA conjugated product (m/z: 1140.3889) corresponds to the calculated mass.

(C) The measured m/z of 3-oxo-Δ^4^-CDCA is very similar to the calculated (m/z: 389.2671 measured, m/z: 389.2686 calculated) and the product after BaiB incubation (3-oxo-Δ^4^-CDCA-CoA) has identical measured and calculated m/z (1138.3733)

(D) Only the chromatogram of 7-oxoLCA after incubation with BaiB is shown. The measured mass of the observed peak corresponds to the calculated mass of 7-oxoLCA-CoA (m/z: 1140.3889). No leftover 7-oxoLCA was found likely due to dilution of the sample.

(E) The measured m/z of 3,7-dioxoCDCA corresponds to the calculated (calculated m/z: 389.2686 vs. measured m/z: 389.2685). The CoA product of 3,7-dioxoCDCA has an identical mass to the calculated (m/z:138.3733).

**Figure S10. Difference in theoretical mass and measured mass from data collected in same LC-MS run.** The difference (in ppm) between the measured mass and the theoretical mass for three internal standards (CDCA-d_4_, DCA-d_4_ and LCA-d_4_) in all samples were plotted in the order of injection into the LC-MS. Most samples were within ± 6 ppm of the theoretical mass.

**References**

1. Marion, S. *et al.* Biogeography of microbial bile acid transformations along the murine gut. *J. Lipid Res.* **61**, 1450–1463 (2020).

2. Vico-Oton, E. *et al.* *Strain-dependent induction of primary bile acid 7-dehydroxylation by cholic acid*. http://biorxiv.org/lookup/doi/10.1101/2022.02.15.480494 (2022) doi:10.1101/2022.02.15.480494.

3. MORRIS, G. N., WINTER, J., CATO, E. P., RITCHIE, A. E. & BOKKENHEUSER, V. D. Clostridium scindens sp. nov., a Human Intestinal Bacterium with Desmolytic Activity on Corticoids. *Int. J. Syst. Evol. Microbiol.* **35**, 478–481 (1985).

4. Hylemon, P. B., Cacciapuoti, A. F., White, B. A., Whitehead, T. R. & Fricke, R. J. 7 alpha-Dehydroxylation of cholic acid by cell extracts of Eubacterium species V.P.I. 12708. *Am. J. Clin. Nutr.* **33**, 2507–2510 (1980).

5. Lagkouvardos, I. *et al.* The Mouse Intestinal Bacterial Collection (miBC) provides host-specific insight into cultured diversity and functional potential of the gut microbiota. *Nat. Microbiol.* **1**, 1–15 (2016).

6. Choi, K.-H. *et al.* A Tn7-based broad-range bacterial cloning and expression system. *Nat. Methods* **2**, 443–448 (2005).

7. Sabri, S., Steen, J. A., Bongers, M., Nielsen, L. K. & Vickers, C. E. Knock-in/Knock-out (KIKO) vectors for rapid integration of large DNA sequences, including whole metabolic pathways, onto the Escherichia coli chromosome at well-characterised loci. *Microb. Cell Factories* **12**, 60 (2013).

8. Jensen, S. I., Lennen, R. M., Herrgård, M. J. & Nielsen, A. T. Seven gene deletions in seven days: Fast generation of Escherichia coli strains tolerant to acetate and osmotic stress. *Sci. Rep.* **5**, 17874 (2015).

9. Boysen, A., Møller-Jensen, J., Kallipolitis, B., Valentin-Hansen, P. & Overgaard, M. Translational Regulation of Gene Expression by an Anaerobically Induced Small Non-coding RNA in Escherichia coli. *J. Biol. Chem.* **285**, 10690–10702 (2010).

10. Durand, S. & Storz, G. Reprogramming of anaerobic metabolism by the FnrS small RNA. *Mol. Microbiol.* **75**, 1215–1231 (2010).
